# Transcriptional and epigenetic changes during tomato yellow leaf curl virus infection in tomato

**DOI:** 10.1186/s12870-023-04534-y

**Published:** 2023-12-18

**Authors:** Beatriz Romero-Rodríguez, Marko Petek, Chen Jiao, Maja Križnik, Maja Zagorščak, Zhangjun Fei, Eduardo R. Bejarano, Kristina Gruden, Araceli G. Castillo

**Affiliations:** 1 Instituto de Hortofruticultura Subtropical y Mediterránea “La Mayora” (IHSM “La Mayora”), Universidad de Málaga-Consejo Superior de Investigaciones Científicas (UMA-CSIC), Boulevard Louis Pasteur, 49, Málaga, 29010 Spain; 2 Department of Biotechnology and Systems Biology, National Institute of Biology, Večna Pot 111, 1000 Ljubljana, Slovenia; 3 Boyce Thompson Institute, Cornell University, Ithaca, NY USA; 4 The Key Lab of Molecular Biology of Crop Pathogens and Insects of Ministry of Agriculture, The Key Laboratory of Biology of Crop Pathogens and Insects of Zhejiang Province, Institute of Biotechnology, Zhejiang University, Hangzhou, 310058 China

**Keywords:** Geminivirus, TYLCV, Tomato, Transcriptome, miRNA, phasiRNA, DNA methylation, Epigenome, Gene silencing, Immune system

## Abstract

**Background:**

Geminiviruses are DNA plant viruses that cause highly damaging diseases affecting crops worldwide. During the infection, geminiviruses hijack cellular processes, suppress plant defenses, and cause a massive reprogramming of the infected cells leading to major changes in the whole plant homeostasis. The advances in sequencing technologies allow the simultaneous analysis of multiple aspects of viral infection at a large scale, generating new insights into the molecular mechanisms underlying plant-virus interactions. However, an integrative study of the changes in the host transcriptome, small RNA profile and methylome during a geminivirus infection has not been performed yet. Using a time-scale approach, we aim to decipher the gene regulation in tomato in response to the infection with the geminivirus, tomato yellow leaf curl virus (TYLCV).

**Results:**

We showed that tomato undergoes substantial transcriptional and post-transcriptional changes upon TYLCV infection and identified the main altered regulatory pathways. Interestingly, although the principal plant defense-related processes, gene silencing and the immune response were induced, this cannot prevent the establishment of the infection. Moreover, we identified extra- and intracellular immune receptors as targets for the deregulated microRNAs (miRNAs) and established a network for those that also produced phased secondary small interfering RNAs (phasiRNAs). On the other hand, there were no significant genome-wide changes in tomato methylome at 14 days post infection, the time point at which the symptoms were general, and the amount of viral DNA had reached its maximum level, but we were able to identify differentially methylated regions that could be involved in the transcriptional regulation of some of the differentially expressed genes.

**Conclusion:**

We have conducted a comprehensive and reliable study on the changes at transcriptional, post-transcriptional and epigenetic levels in tomato throughout TYLCV infection. The generated genomic information is substantial for understanding the genetic, molecular and physiological changes caused by TYLCV infection in tomato.

**Supplementary Information:**

The online version contains supplementary material available at 10.1186/s12870-023-04534-y.

## Background

Tomato (*Solanum lycopersicum*) is one of the most important fruit or vegetable crops worldwide and a model research plant. Tomato genomes sequenced in the past decade provided a wealth of data that facilitates gene characterization of this agronomically important plant [1–3]. Among the main threats affecting the world production of this plant are losses due to viral infections [4]. Understanding the molecular and cellular mechanisms underlying the interaction of tomato and viruses is essential for developing effective strategies to manage the infections.

Geminiviruses are plant viruses with single-stranded DNA (ssDNA) circular genomes, transmitted by phloem-feeding insects that cause highly damaging diseases affecting food, feed, and fiber crops worldwide. The *Geminivirideae* family is classified into 14 different genera, including *Begomovirus*, the largest genus of plant-infecting viruses with more than 440 species [5]. It is the only geminiviral genus transmitted by the polyphagous whitefly *Bemisia tabaci* (Family *Aleyrodidae*), which is considered one of the main vector species of plant viruses [6, 7]. The begomovirus genome can be either bipartite (two circular ssDNA molecules independently encapsidated) or monopartite (a single circular ssDNA molecule). The small size of these genomes (around 2.7 kb) imposes a constraint on coding capacity, but the evolution of overlapping open reading frames (ORFs) that encode four to nine proteins has partially compensated for this size limitation [8]. *Tomato yellow leaf curl virus* (TYLCV) is a monopartite begomovirus that encodes nine proteins. TYLCV is the principal causing agent of Tomato yellow leaf curl disease (TYLCD), which is a viral disease that affects several crops from the *Solanaceae* family, such as tomato, pepper and eggplant [9–12].

During the infection, the geminiviral proteins interfere with the cellular machinery, causing a massive reprogramming of the infected cells that leads to significant changes in the whole plant homeostasis allowing them to seize the plant machinery required for the viral cycle and impairing the antiviral defenses [8, 13, 14].

RNA silencing, also known as RNA interference (RNAi), is the main antiviral defense mechanism in plants, with viruses acting as both inducers and targets [15, 16]. Inside the host cells, the geminiviral ssDNA is converted into double-stranded forms, which associates with nucleosomes to form minichromosomes that are subjected to transcriptional gene silencing (TGS). In addition to TGS, the RNA silencing machinery responds to geminiviral infection, triggering post-transcriptional gene silencing (PTGS), which is directed against the viral transcripts. To counteract these host silencing-based antiviral mechanisms, geminiviruses encode silencing-suppressor proteins (e.g. several TYLCV proteins act as PTGS (TrAP, C4, V2, V3, C5, C7) or TGS suppressors (TrAP, Rep, V2, V3, C5) [8, 10–12, 17, 18].

The plant immune system is based on pattern recognition receptors (PRRs) at the plasma membrane, such as Receptor Like Kinases (RLKs) and Receptor Like Proteins (RLPs), that perceive pathogen-associated molecular patterns (PAMPs) or plant-derived damage-associated molecular patterns (DAMPs). The ligand perception is transduced into intracellular signaling to induce a Pattern Triggered Immunity (PTI) response. An additional but interconnected branch of the immune response is the Effector Triggered Immunity (ETI) which is mediated by the recognition of pathogen-derived molecules by intracellular receptors known as Nucleotide-binding Leucine-rich repeat Receptors (NLRs) [19–21]. The role of ETI in antiviral defense through resistance proteins (NLRs) has been well documented for RNA and DNA viruses [22, 23]. Two NLRs of the coil-coiled type, *Ty-2* and *Sw5a*, have been described as resistance loci in tomato for TYLCV [24] and *Tomato leaf curl New Delhi virus* (ToLCNDV) [25], respectively. Although the implication of PTI in viral defense is less documented, several data indicate that PTI extracellular receptors could play a role in restricting viral infection [26–29]. Several geminiviral proteins have been shown to interact with RLKs [30], including NUCLEAR SHUTTLE PROTEIN-INTERACTING KINASE 1 (NIK1), a RLK which is involved in antiviral defense, since its deletion increases susceptibility to infection, and its overexpression confers tolerance to begomovirus infection [26, 28, 31].

The use of omics technologies has profoundly impacted the study of plant-virus interactions, allowing researchers to gain a more comprehensive understanding of the complex molecular interactions that occur during viral infection. A remarkable number of studies have used high-resolution genome-wide sequencing to analyze the modifications in the transcriptional landscape of the host upon viral infection [32]. Since the first comprehensive transcriptome analysis of *Arabidopsis thaliana* plants infected with a geminivirus [33], the transcriptional changes triggered by geminivirus infection have been characterized in several plant species, including crops such as cassava, mung bean, melon, cotton, and various species from the *Solanaceae* family [34–43]. However, in general, few commonalities are detected if their results are compared, probably due to the differences in the experimental conditions and design and /or the timing for tissue sampling.

The expression of host small RNAs (sRNAs) during geminivirus infection is less characterized than that of the mRNA transcriptome. Most studies focus on a specific microRNA (miRNA) and its targets, or on miRNAs that regulate a specific cellular process [25, 44–47]. By this approach some miRNAs involved in tomato-ToLCNDV interaction, such as sly-miR159 and sly-miR166c, have been identified [25, 47]. However, to the best of our knowledge, the host genome-wide sRNA landscape changes during a geminiviral infection have not yet been characterized.

Geminiviral proteins affect the functioning of the cellular methyl cycle and interfere with the host DNA methylation machinery at different steps. Viral proteins, such as TrAP, Rep and V2, are able to interfere with the DNA methylation levels at certain host loci (mainly transposons (TEs) or repeats) or transgenes that are transcriptionally silenced [17, 18, 48–53]. Although an early attempt using methylation-sensitive amplification polymorphism showed changes in DNA methylation levels at certain tomato loci during the infection with the begomovirus *Tomato yellow leaf curl Sardinia virus* (TYLCSV) [54], a single nucleotide resolution analysis of the tomato methylome upon geminiviral infection is still needed. Despite the numerous genome-wide analyses carried out to study plant-geminivirus interactions, no integrated study of mRNA, sRNA, and methylome profiling along the different stages of the infection is available. Using short-read sequencing, we have followed the changes in the mRNA and sRNA transcriptomes of TYLCV-infected tomato plants at four time points (2, 7, 14, and 21 days post-inoculation, dpi), as well as the tomato methylome at 14 dpi by whole-genome bisulfite sequencing (WGBS). Analysis and integration of these data provided an overview of the changes in host gene expression as well as its dependency on sRNA regulation (miRNA and phased secondary small interfering RNAs (phasiRNA)) and DNA methylation during TYLCV infection. This study represents the first comprehensive analysis of the changes in tomato mRNA and sRNA transcriptome and methylome upon a geminivirus infection.

## Results

### Transcriptional changes during TYLCV infection in tomato plants

Tomato plants were infected with TYLCV by agroinoculation. As controls, plants were exposed to *Agrobacterium tumefaciens* carrying a binary plasmid (mock) or were non-treated (naïve plants) (Fig. S1). Symptom development was measured using a semi-quantitative scale, in which 0 corresponds to no symptoms and 5 to the most severe symptoms including curling and yellowing of the leaflets and inhibition of plant growth. Mild TYLCV symptoms appeared in some plants at 7 dpi, while at 14 dpi, all inoculated plants displayed typical TYLCV severe symptoms that became more intense at 21 dpi (Fig. S2A).

Apical leaf tissue was collected at 2, 7, 14, and 21 dpi and DNA was extracted to quantify the accumulation of total viral DNA. In accordance with symptom development, viral DNA was not detected at 2 dpi but at 7 dpi. The amount of viral DNA increased markedly by 14 dpi and remained at similar levels one week later (21 dpi) (Fig. S2B). RNA was extracted from the same samples and RNA-seq was performed by Illumina sequencing (36 libraries; three biological replicates of naïve, mock and TYLCV-infected plants at the four time points). We obtained between 27 and 64 million raw pair-end reads (Mr) per sample, with an average of 33.7 Mr, and the mapping rate to the tomato genome ranged from 93 to 95% (Dataset S1). In a prior publication, the transcription of viral genes was thoroughly examined by mapping the cleaned reads from infected samples to the TYLCV genome [55].

**Fig. 1:**
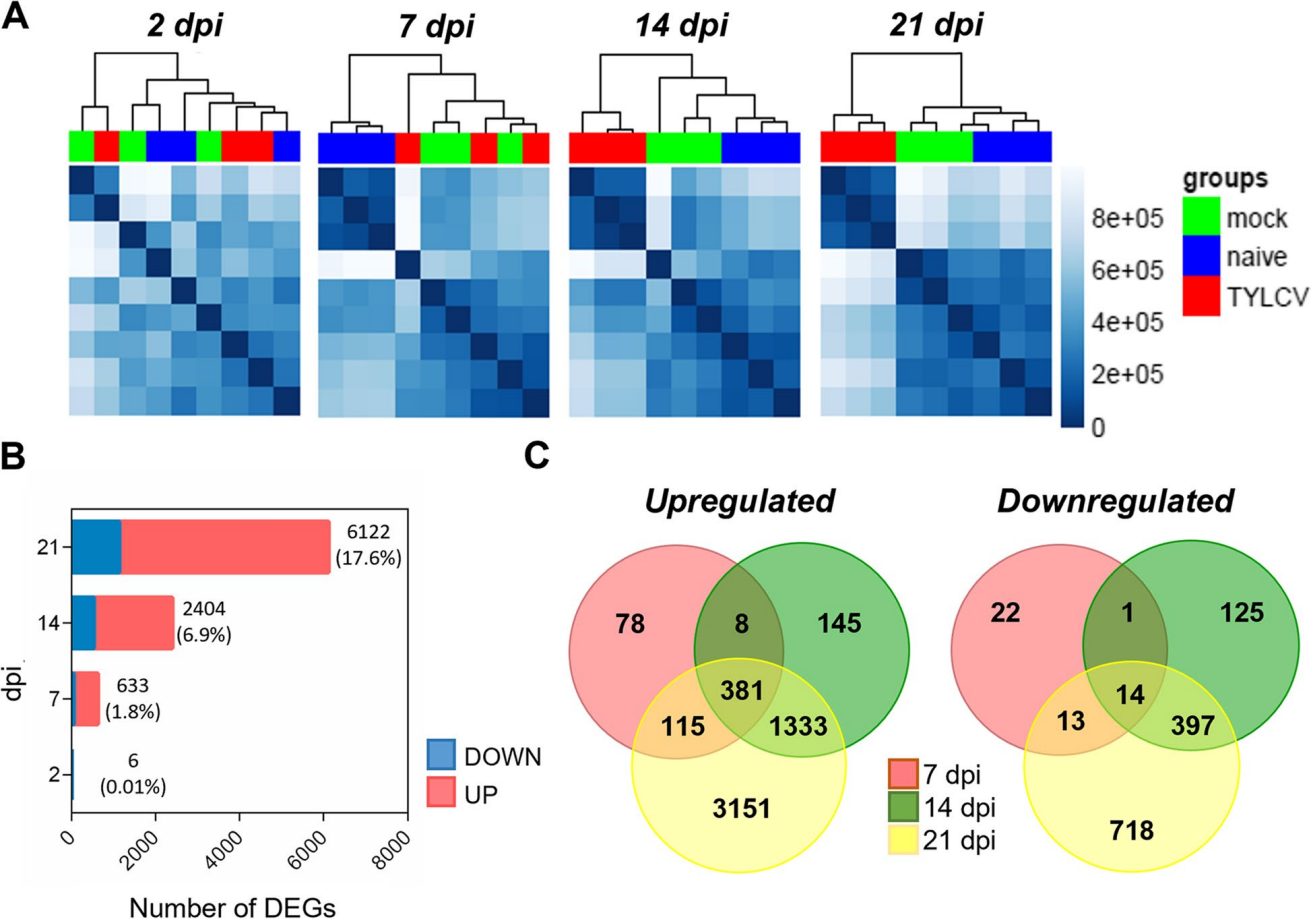
Transcriptional changes during TYLCV infection in tomato plants. **A** Gene Expression profiles of tomato genes in naïve (blue), mock (green) and TYLCV-infected samples (red) at 2, 7, 14 and 21 dpi are shown in heatmaps with hierarchical clustering. The blue color bar on the right indicates the normalized read counts. **B** Stacked bar charts showing the numbers of upregulated (red) and downregulated (blue) DEGs comparing TYLCV-infected versus mock samples during TYLCV infection at 2, 7, 14 and 21 dpi. **C** Venn diagram showing common and specific upregulated and downregulated DEGs at 7, 14 and 21 dpi. For B) and C) only DEGs with FDR adjusted *p*-value ≤ 0.05 and ≥ 1.5-fold induction or ≤ 0.75-fold repression, were represented

Hierarchical clustering of the transcriptomes showed that at 2 dpi infected and non-infected (naïve and mock) samples cluster together, indicating that there were no major changes in the tomato transcriptome at that time point (Fig. 1A). Although TYLCV DNA was detected systemically at the apical leaves at 7 dpi (Fig. S2B), the changes in the tomato transcriptional landscape were limited, as mock and TYLCV-infected samples arranged together in the hierarchical clustering analysis whereas naïve samples clustered separately. This suggests that in addition to TYLCV, a substantial part of the transcriptome changes in the infected plants at 7 dpi were due to the presence of *Agrobacterium* (Fig. 1A). However, at 14 dpi, the TYLCV-infected samples clustered separately from naïve and mock samples and this pattern was maintained at 21 dpi, showing extensive changes in tomato transcriptome upon TYLCV infection (Fig. 1A). When comparing TYLCV-infected versus mock samples, the number of differentially expressed genes (DEGs) increased during the systemic infection, ranging from 6 DEGs at 2 dpi to 6122 DEGs at 21 dpi (Fig. 1B). The number of DEGs at 7 dpi represented 1.8% of tomato genes (considering 34,727 tomato genes [1]) and this amount increased almost fourfold at 14 dpi (6.9%) when viral DNA accumulation had reached a *plateau*. Although the amount of viral DNA was maintained from 14 to 21 dpi (Fig. S2B) the number of DEGs increased more than twofold during this time frame, indicating that there were transcriptional changes in the host, even though the amount of viral DNA did not significantly change (Fig. 1B). Most of the DEGs were upregulated during TYLCV infection, with a large proportion of them consistently maintaining their overexpression throughout time. Sixty-five percent (383/582) of the genes that were induced at 7 dpi remained upregulated at 14 and 21 dpi and 92% (1714/1867) of the genes overexpressed at 14 dpi, stayed induced at 21 dpi. Interestingly 20% (115/582) of the genes that were induced at 7 dpi were also upregulated at 21 dpi but not at 14 dpi (Fig. 1C). For the downregulated genes the scenario was different as only 27% (14/51) of the repressed genes at 7 dpi, remained downregulated throughout the infection, and a similar percentage (25,5%, 13/51) was repressed at 7 and 21 dpi but not at 14 dpi. On the other hand, most of the downregulated genes at 14 dpi (76.5%, 411/537) stayed repressed one week later (21 dpi) (Fig. 1C).

### Functional enrichment of tomato DEGs in response to TYLCV infection

**Fig. 2:**
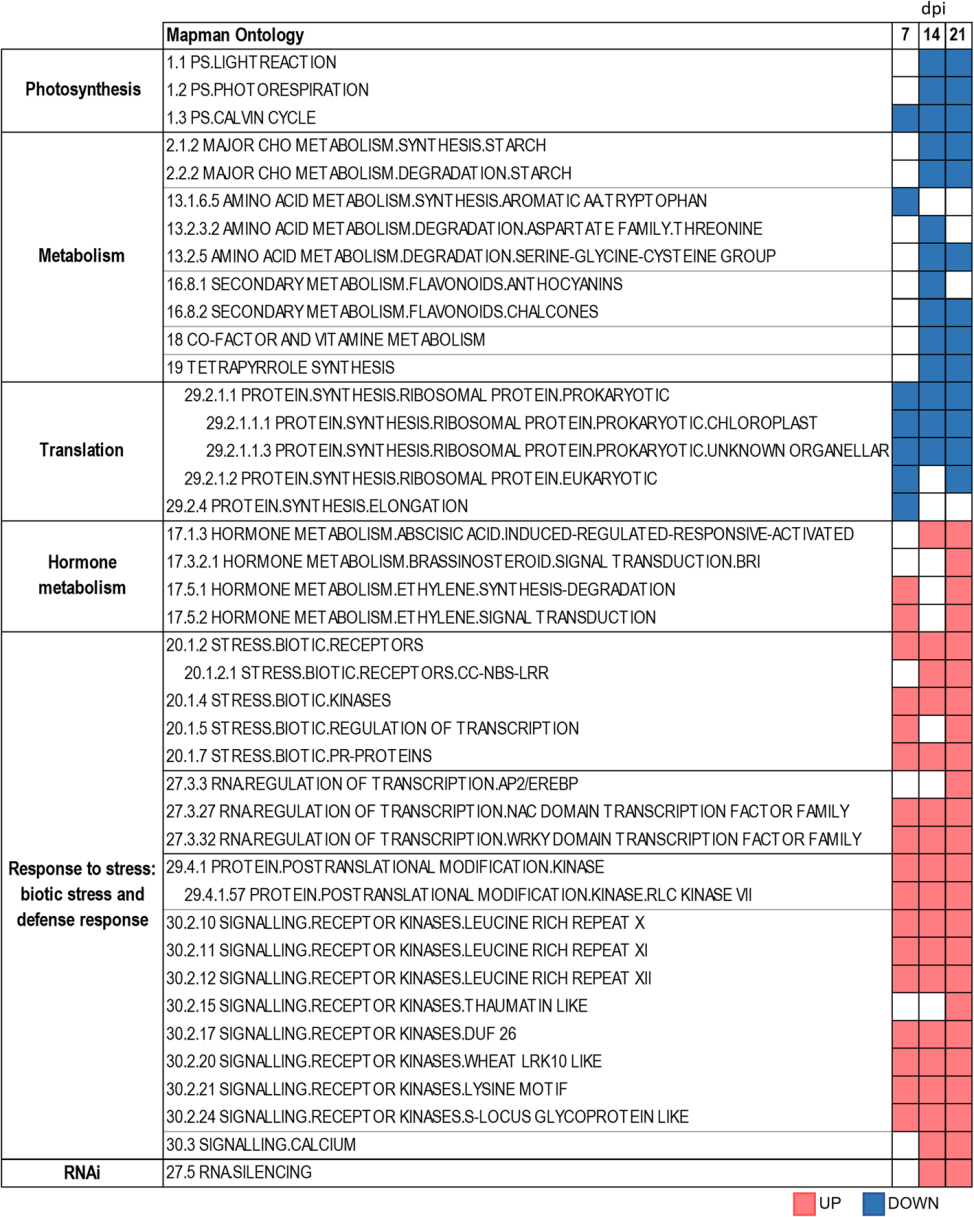
Biological processes transcriptionally deregulated in tomato in response to TYLCV infection. Gene Set Enrichment Analysis (GSEA) computational method and MapMan ontology as the source for the gene sets were used to identify the biological processes significantly enriched in upregulated (red-colored) or downregulated genes (blue-colored) at 7, 14 and 21 dpi. No color indicates no statistically significant enrichment (FDR adjusted *p*-value ≤ 0.05)

We identified the biological processes significantly enriched in up- or downregulated genes during TYLCV infection using the Gene Set Enrichment Analysis (GSEA) computational method [56] with the MapMan ontology [57]. Among repressed genes, we observed a significant enrichment of those related to translation, primary (carbohydrates and amino acids) and secondary (flavonoids) metabolism and photosynthesis (Fig. 2, Dataset S2). On the other hand, we observed a significant enrichment of upregulated genes associated with biotic stress, defense response, RNAi, and hormone responses. It is worth mentioning that most of the processes induced at 21 dpi, were already induced at 14 dpi and many of them also at 7 dpi. An interesting exception is the ethylene-mediated response that was induced at the beginning of the infection and at 21 dpi, but not at 14 dpi (Fig. 2, Dataset S2). The analysis of the Gene ontology (GO) functional enrichment of the DEGs in the biological processes category resulted in the identification of similar categories to the GSEA for the upregulated and downregulated genes. However, using GO functional enrichment analysis we identified additional biological processes over-represented for induced genes at 21 dpi, such as autophagy, vesicle-mediated transport, or ubiquitin-dependent protein catabolic process via the multivesicular body sorting pathway (Fig. S3).

A closer look at the “biotic stress” and “defense response” categories at 14 and 21 dpi using MapMan, revealed that many of the subcategories related to the plant immune response, including plasma membrane pattern recognition receptors (RLKs and RLPs), Receptor Like Cytoplasmatic Kinases (RLCKs), intracellular receptors (NLRs), transcription factors, and pathogenesis-related proteins (PR), were overrepresented in upregulated genes (Fig. S4A to S4F). Based on the nature of the ligand-binding extracellular domain of the RLKs/RLPs, they are divided into different subfamilies and several of them were overrepresented with upregulated genes: LRR (Leucine-Rich Repeat), DUF26 (Domain of Unknown Function 26), LRK10-like (Leaf Rust 10 Disease-Resistance Locus Receptor-Like Protein Kinase), S-locus (Self-incompatibility locus), and Thaumatin (Fig. S4A and S4B). The LRR-RLK constitutes the largest subfamily (around 38% of tomato RLKs) and it is divided into 15 groups (from I to XV) [58, 59], three of which (X, XI and XII) were overrepresented with upregulated genes at both time points (Fig. S4B). Although most RLKs are localized in the plasma membrane, there is a large RLK subfamily named RLCK with 128 members in tomato, that does not possess either an extracellular region or a transmembrane domain. Interestingly, the group VII of the RLCKs, which contains members that mediate PTI and contribute to resistance against bacterial and fungal pathogens as well as aphids [60–64], was the only RLCK group overrepresented with upregulated genes at 14 and 21 dpi (Fig. S4C). The intracellular NLRs involved in ETI, were also significantly induced upon TYLCV infection (Fig. 2, Fig. S4D). Furthermore, at 14 and 21 dpi the MapMan BIN holding transcription factors which are involved in biotic stress responses, such as WRKY, DOF (DNA-binding One Zinc Finger) and ERF (Ethylene Responsive Factor), and the PR genes were also overrepresented in upregulated genes (Fig. 2, Fig. S4E and S4F). This suggests that TYLCV infection induced the transcriptional reprogramming of the host to establish the plant immune response. Additionally, the categories that included genes involved in hormone-dependent responses, such as jasmonic acid (JA), ethylene, and abscisic acid (ABA), were also significantly enriched in upregulated genes as response to the infection (Fig. 2, Fig. S4G).

The ontology category that included the main defense mechanism against plant viruses, i.e., RNA silencing, was also overrepresented in upregulated genes in the GSEA and MapMan analyses (Fig. 2, Dataset S2). To characterize this response in more detail, we examined the expression of the main Arabidopsis orthologues of *DCL* (*DICER-like*), *AGO* (*ARGONAUTE*) and *RDR* (*RNA-dependent RNA polymeras*e) genes. The expression of the core RNA silencing machinery genes increased steadily after the infection, reaching the highest levels at 21 dpi (Table 1).

**Table 1:**
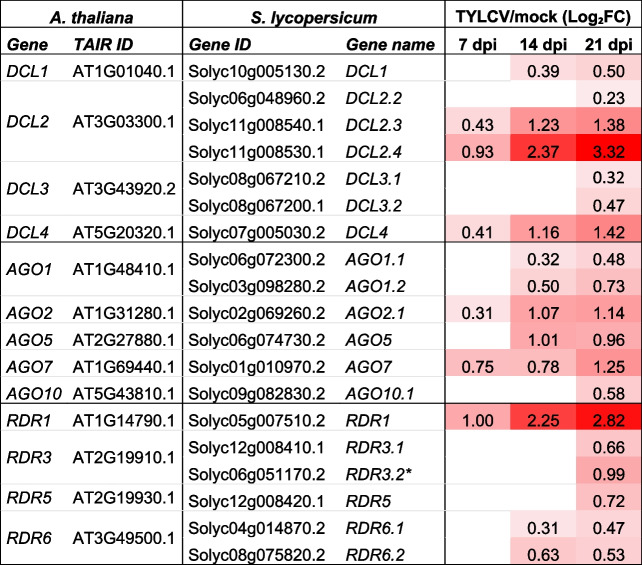
Differentially expressed tomato genes involved in post-transcriptional gene silencing during TYLCV infection

^*^RDR3.2 (Solyc06g051170.2) correspond to the Ty-1/Ty-3 resistance gene

### Dynamics of the transcriptional changes during TYLCV infection

**Fig. 3:**
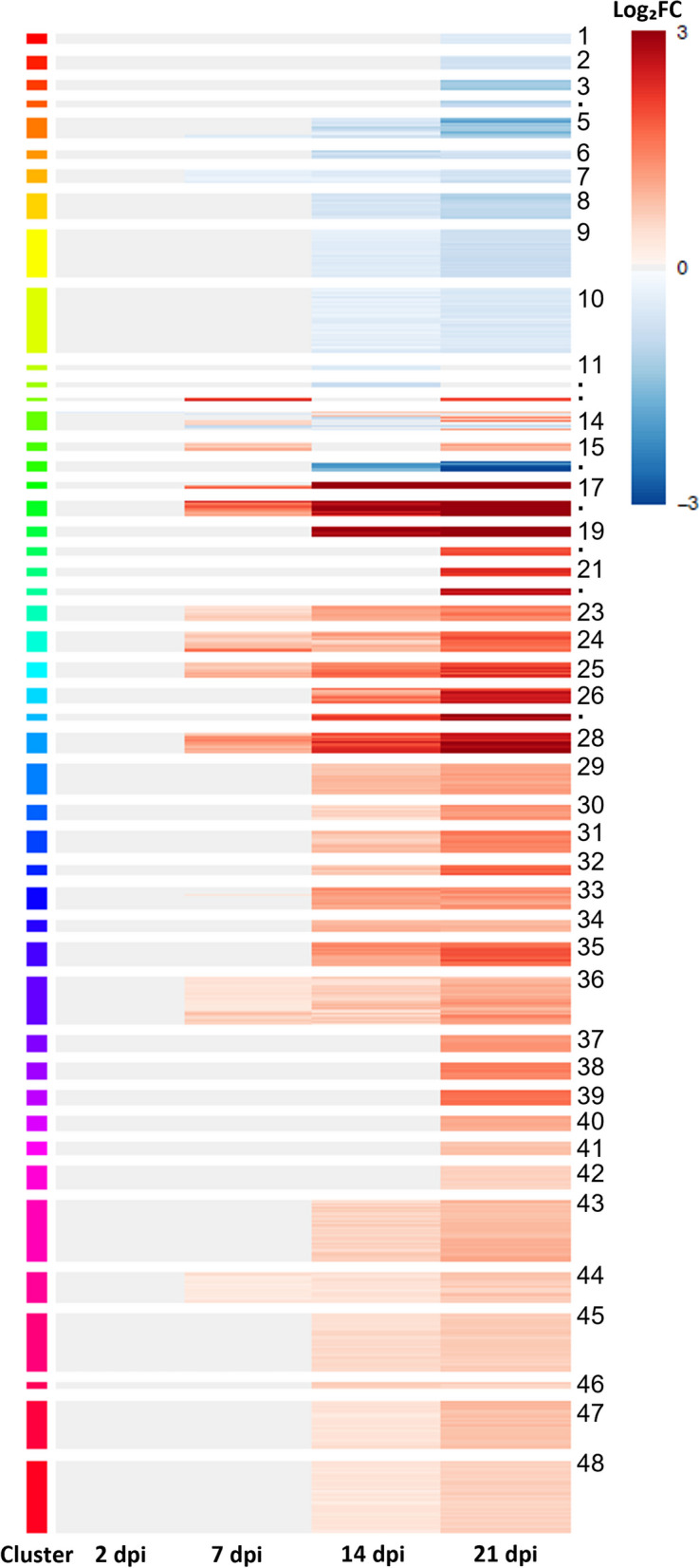
Dynamics of transcriptional regulation during TYLCV infection in tomato plants. Clustering of DEGs in tomato plants in response to TYLCV infection (2, 7, 14 and 21 dpi) using SplineCluster algorithm. Cluster membership is shown by colors on the left and numbered on the right (missing cluster numbers are represented by dots). Expression of transcripts in clusters is presented as heatmap. The color scale for gene expression on the right, red to blue, represents highly positive to highly negative log_2_FC (FC: ratio TYLCV/mock). Gray indicates not differentially expressed genes

To better understand the genome-wide transcriptional changes upon TYLCV infection in a time-dependent manner, we performed a cluster analysis of the DEGs and obtained 48 clusters. The upregulated genes were comprised in 32 clusters while the downregulated ones in 13 (Fig. 3, Fig. S5, Dataset S3). A few genes included in tree clusters (11, 12 and 13), changed from induced to repressed or vice versa along the infection; however, no obvious biological or functional relations between these genes were found.

As mentioned before, most genes that were induced at early stages of infection (7 dpi) stayed deregulated up to 21 dpi (Fig. 3, Fig. S5). GO enrichment analysis of those genes, identified functional categories mainly related to defense response and gene silencing (Fig. S6A). When MapMan analysis was performed on those genes, overrepresented terms were related to genes encoding stress biotic receptors, mainly NLR genes and signaling receptor kinase genes, including LRR-RLKs (groups XI and XII) (Fig. S6B). This analysis indicated that the first antiviral response from the plant was a wave of induced genes that was maintained throughout the infection and comprised the two main plant defense mechanisms: gene silencing and the immune response. A second wave of upregulated genes at 14 dpi which stayed induced at 21 dpi, belonged to other GO categories related to response to pathogens, such as: (i) protein autophosphorylation of calcium dependent protein kinases [65, 66], (ii) protein N-linked glycosylation, which plays a relevant role in plant immunity against pathogens and pest [67, 68], (iii) signal transduction, including RLKs and (iv) localization, which encompasses components of the vesicle trafficking pathway (Fig. S6C). On the other hand, there were fewer genes repressed in response to TYLCV infection and most of them were deregulated at 14 dpi and stayed repressed at 21 dpi (Fig. 3). Functional enrichment analysis of these late infection-repressed genes identified overrepresented terms such as photosynthesis, glycolytic process, plastid organization, alpha-amino acid metabolic process and cellular response to oxidative stress (Fig. S6D).

### Tomato small RNA profile during TYLCV infection

TYLCV infection interfere with the proper functioning of the plant gene silencing pathway by the production of viral suppressors of RNAi (VSRs) and the generation of large amounts of viral RNAs and sRNAs that could “overflow” the RNAi machinery [55, 69]. To assess the impact of TYLCV on gene silencing regulation in tomato, we analyzed the host small RNA (sRNA) profile during the viral infection. Deep sequencing of sRNA libraries (24 in total from naïve, mock and TYLCV-infected samples) was performed on two of the three biological replicates used to resolve the transcriptome at 2, 7, 14 and 21 dpi (Fig. S1, Dataset S1). The total number of raw sRNA reads ranged from 64 to 92 million (81 high-quality million reads on average per sample, Dataset S1). The normalized amount of total cleaned 18–26-nt sequences that were mapped to tomato genome discarding the reads from other non-coding RNAs (rRNAs, tRNAs, snRNAs and snoRNAs), ranged from 82 to 89% (average 86%, Dataset S4). The cleaned reads from infected samples were also mapped to the TYLCV genome, and the characterization of the viral sRNA landscape during the infection was previously described [55].

**Fig. 4:**
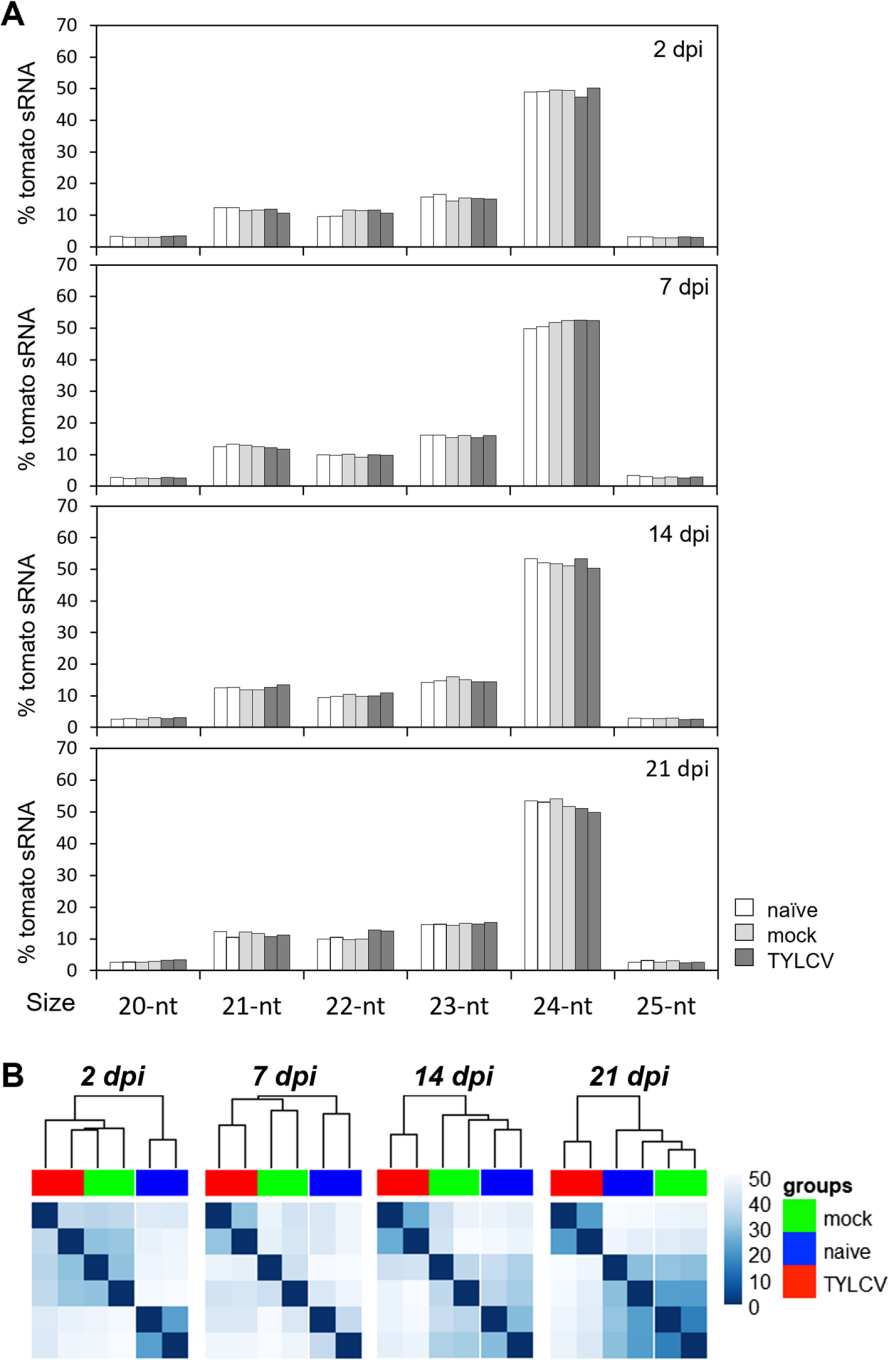
Tomato sRNA profile during TYLCV infection. **A** Percentage of each size-class of 20–25-nt sRNA reads relative to the total sRNA reads that mapped to the tomato genome (18–26-nt) in naïve, mock and TYLCV-infected tomato samples at 2, 7, 14 and 21 dpi. Each bar corresponds to one biological replicate. **B** Expression of tomato sRNAs in naïve (blue), mock (green) and TYLCV infected samples (red) at 2, 7, 14 and 21 dpi are shown in heatmaps with hierarchical clustering. The blue color bar on the right, indicates the normalized read counts per million (CPM)

The analysis of the accumulation and size distribution of the tomato sRNAs, showed that in agreement with previous data for tomato leaves [70, 71], the 24-nt sRNAs were the most abundant size class (51%) followed by 21-, 22- and 23-nt sRNAs that accumulate in similar quantities (10%, 12% and 16%, respectively) (Fig. 4A, Dataset S4). No significant changes in the overall tomato sRNA size distribution between naïve, mock and infected samples could be detected throughout TYLCV infection (Fig. 4A).

Hierarchical clustering analysis of the total tomato sRNA population from naïve, mock, and TYLCV-infected samples showed that at 14 dpi, sRNAs from TYLCV-infected tissues clustered separately from mock and naïve samples similarly to the gene expression data. This distribution was maintained at 21 dpi as well (Fig. 4B).

We identified and quantified the differentially expressed siRNA loci (DEsiRNAs) of 21-, 22- and 24-nt throughout TYLCV infection and mapped them to gene bodies and promoters (defined as 2 kb upstream the transcriptional start site) of tomato protein-coding genes, and to TEs/repeats. The number of DEsiRNAs was similar at 7 and 14 dpi but larger at TEs/repeats than at gene bodies or promoters (Fig. S7, Datasets S5-S10). However, there was a significant increase in the number of DEsiRNAs from 14 to 21 dpi, mainly due to the increase in the number of differentially expressed 24-nt hetsiRNAs (29-fold for those at gene bodies, 22-fold at promoters and 13-fold at TEs/repeats) (Fig. S7, Datasets S5-S10).

### Changes in the microRNA expression profile in tomato during TYLCV infection

MicroRNAs (miRNAs) are key regulators of cellular homeostasis and are involved in many essential cellular processes, including cell defense responses [72, 73]. Transcription of *MIR* genes results in generation of hairpin miRNA transcripts, that are processed by the RNA silencing machinery, typically producing 21- or 22-nt mature miRNAs [74]. The miRNAs detected in our infected and control samples at any time point represented approximately 7% of the total sRNA fraction that could be assigned to the tomato genome (Dataset S4). To examine the miRNA population in more detail, we first compared the miRNA sequences with annotated miRNAs in miRBase database and identified 135 unique tomato miRNA that belonged to 37miRNA families and two unique miRNAs that belong to a new family (sly-miR1-5p) (Table 2, Fig. S8, Dataset S11). Around 28.2% of these miRNAs were conserved in other plant species, 9.6% were solanaceous-specific, and 13.3% were *S. lycopersicum*-specific. Moreover, we found unique unannotated miRNAs (46.4%) that belonged to 22 known families (miRNA isoforms) and 2 unannotated miRNAs that belong to the new sly-miR1-5p family (Table 2, Dataset S11) [75, 76]. The sly-miR166 family was the one with the highest number of expressed miRNAs in our dataset (Fig. S8, Dataset S11).

**Table 2:** Classification of tomato miRNAs identified in the sRNA-seq dataset

	***Unique***	***Families***
**Annotated miRNA**		
Conserved	38	14
*Solanaceae*-specific	13	10
*S. lycopersicum*-specific	18	17
**Unannotated miRNA**		
Known family	64	22
New family	2	1^a^

^*a*^* novel-sly-miR1-5p*

**Fig. 5:**
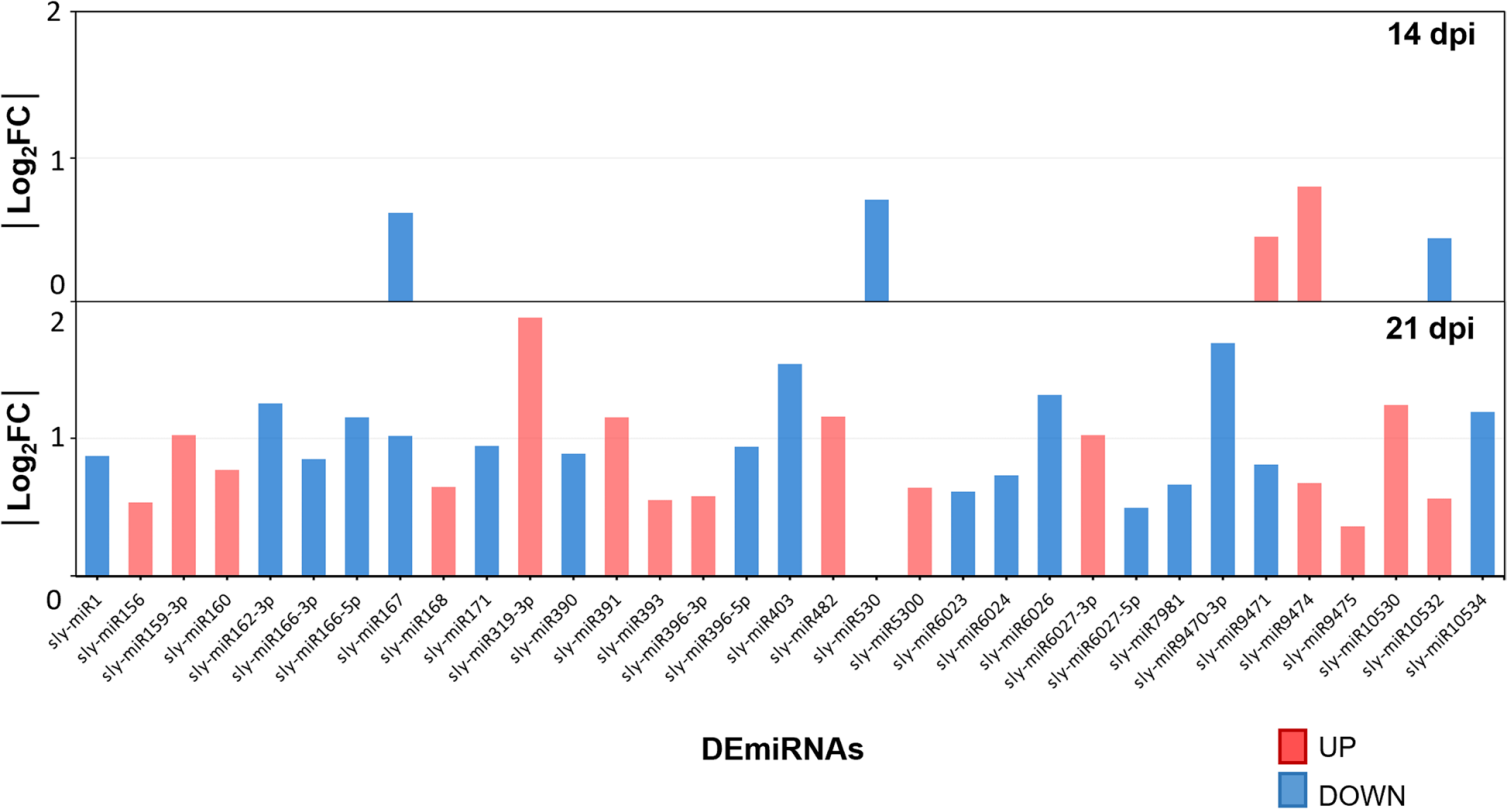
Tomato miRNA expression changes during TYLCV infection. miRNA expression changes (|log_2_FC|, absolute value of the log_2_FC) in TYLCV-infected samples compared with mock-infected at 14 and 21 dpi. Upregulated miRNAs are shown in red and downregulated ones in blue

The comparison of the miRNA expression between infected and mock samples let us identify the differentially expressed miRNAs (DEmiRNAs) during TYLCV infection. We could not detect any DEmiRNAs at 2 dpi or even at the onset of the symptoms at 7 dpi, when the transcriptional changes in tomato have already started (Fig. 1A). At 14 dpi, once the relative levels of viral DNA reached a *plateau* and around 7% of the tomato genes were deregulated (Fig. 1B), only 5 miRNAs were differentially expressed. However, at 21 dpi there was an increase in the number of DEmiRNAs and we identified 32 miRNAs whose expression was deregulated with a similar number of miRNAs induced and repressed (15 and 17, respectively) (Fig. 5, Dataset S11). The comparison among the DEmiRNAs between 14 and 21 dpi showed that one out of the five was repressed just at 14 dpi (sly-miR530), two did not change their behavior (sly-miR167 and sly-miR9474), and the other two (sly-miR9471 and sly-miR10532) were deregulated in the opposite direction at both time points (Fig. 5). Regarding the nature of these DEmiRNAs, a detailed analysis using miRNA databases and published results showed that the tomato miRNAs deregulated upon the infection targeted mainly genes encoding: (i) transcription factors (sly-miR156, sly-miR159-3p, sly-miR166-3p, sly-miR171, sly-miR319-3p, miR396-5p); (ii) transcripts involved in auxin response (sly-miR160, sly-miR167, sly-miR393); (iii) transcripts from actors of the gene silencing machinery (sly-miR168, sly-miR403) and (iv) immune receptors, RLKs and NLRs (sly-miR390, sly-miRNA396-3p, sly-miRNA396-5p, sly-miRNA482, sly-miRNA6023, sly-miRNA6024, sly-miRNA6026, sly-miRNA6027-3p).

### Posttranscriptional regulation of the miRNA target genes during TYLCV infection

**Fig. 6:**
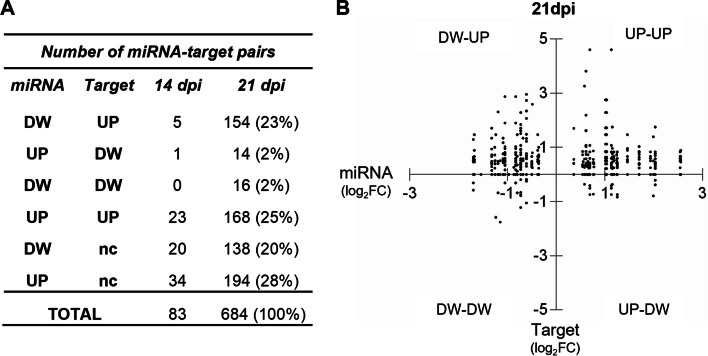
Expression levels of the DEmiRNAs and their predicted target genes in TYLCV-infected tomato plants. **A** Classification of the putative miRNA-target pairs at 14 and 21 dpi based on their expression levels. UP: upregulation, DW: downregulation, nc (no change): target gene is not differentially expressed. **B** Expression level (log_2_FC for the ratio TYLCV/mock) at 21 dpi of the DEmiRNAs (x axis) and their target genes (y axis)

To assess the impact of the deregulation of tomato miRNAs on the accumulation of their target transcripts, we compared the expression levels of the DEmiRNAs and their predicted targets [77, 78]. The number of predicted target genes for the deregulated miRNAs extended to 83 at 14 dpi and to 684 at 21 dpi (Fig. 6A). We checked the expression levels of those predicted target genes in our mRNA transcriptome dataset and at 21 dpi, only 25% of the pairs showed the expected inverse canonical relationship for a miRNA and its target (Fig. 6A and 6B). In 27% of the pairs, miRNAs and the target transcripts were deregulated in the same direction (both induced or repressed) and in almost half of the pairs (48%) there were no changes in the expression of the target genes, although their matching miRNAs were deregulated (Fig. 6A and 6B). This non-canonical pattern of the miRNA-target pairs was also detected at 14 dpi (Fig. 6A) and indicated that upon TYLCV infection, transcriptional regulation was the most important level of regulation and that miRNAs mainly modulates the abundance of transcripts [79–81]. Functional enrichment analysis (GO) of the genes whose transcripts were targets of the DEmiRNAs at 21 dpi, showed that the categories related to plant defense response and morphogenesis/development were overrepresented (Fig. S9A). Similarly, MapMan enriched terms were the ones containing biotic stress receptors (NLRs, among others) and receptor kinases involved in signaling, which included RLKs and RLPs (Fig. S9B), indicating that upon infection, defense response genes were the most abundant targets for the DEmiRNAs.

To validate the miRNA-target pairs predicted from our datasets, we took advantage of the degradome sequencing data generated from leaves of the same tomato variety (Moneymaker) infected with a closely related TYLCV isolate (TYLCV-[CN:SH2], [82]). Four degradome datasets were analyzed with the CleaveLand4 tool [83] using our tomato sRNA-seq and transcriptome sequences. When comparing the levels of expression from the DEmiRNAs and their target transcripts, we found a similar pattern to that previously described above in Fig. 6: the canonical pattern for the expression of the miRNAs and their targets was found in just 39% of the pairs (Fig. S10A and S10B, Dataset S12).

### Comparative expression among phasiRNAs, their predicted target genes and PHAS loci during TYLCV infection

**Fig. 7:**
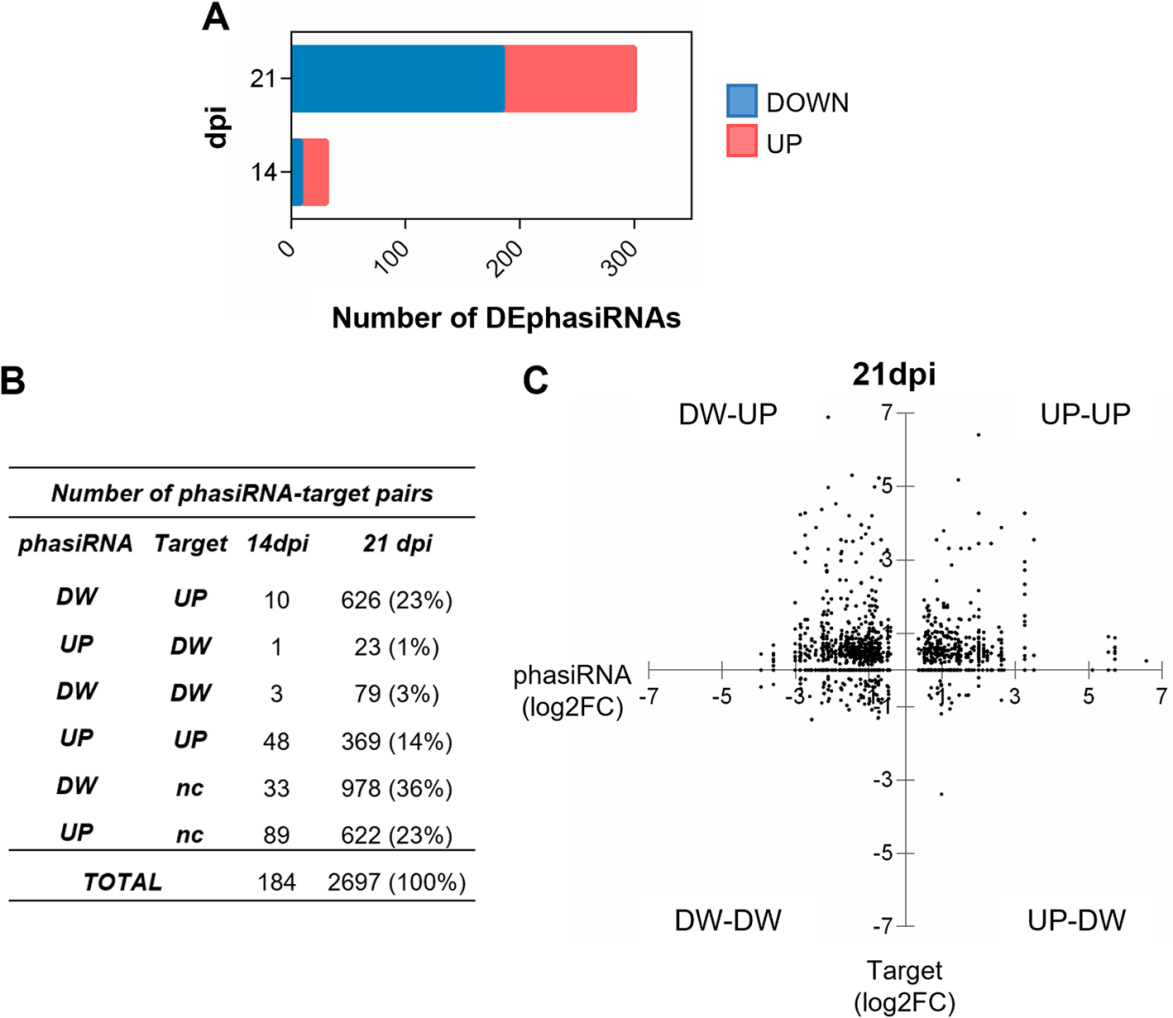
Expression levels of the DEphasiRNAs and their predicted target genes in TYLCV-infected tomato plants. **A** Stacked bar charts showing the number of DEphasiRNAs comparing TYLCV-infected versus mock samples at 14 and 21 dpi, indicating the upregulated (UP, red) and downregulated (DOWN, blue) phasiRNAs. **B** Number of putative phasiRNA-target pairs at 14 and 21 dpi based on the miRNA and the target genes expression levels. UP: upregulation, DW: downregulation, nc (no change): target gene is not differentially expressed. **C** Expression level (log_2_FC for the ratio TYLCV/mock) at 21 dpi of the DEphasiRNAs (x axis) and their target genes (y axis)

Some 22- or 21-nt miRNAs can act as triggers for the biogenesis of phased secondary small interfering RNAs (phasiRNAs) by targeting specific phasiRNA precursor transcripts produced from *PHAS* loci [84]. Using phasiRNA and *PHAS* prediction pipeline, we found more than 12,000 phasiRNAs derived from 799 *PHAS* loci (Dataset S11 and Dataset S13). To investigate the changes in the phasiRNA levels we compared their expression between virus and mock samples and identified the phasiRNAs that were differentially expressed (DEphasiRNAs) during the infection. Like the DEmiRNAs, very few DEphasiRNAs were detected at the beginning of the infection (none at 2 dpi and 3 at 7 dpi). The number of DEphasiRNAs increased later in the infection from 31 to 300 at 14 and 21 dpi, respectively (Fig. 7A, Dataset S14). The number of predicted target genes for the deregulated phasiRNAs at 21 dpi reached more than two thousand genes (2697) (Fig. 7B). Similarly, as observed for the miRNA-target pairs (Fig. 6), the correlation between the expression levels of the deregulated phasiRNAs and their predicted target genes was weak (Fig. 7B and 7C). Functional enrichment of the putative target genes for the DEphasiRNAs showed an overrepresentation of terms related to defense response (including biotic stress receptors, NLRs, and several families of RLKs), gene silencing, cell cycle, cell wall biogenesis and vacuolar acidification and localization (vesicle trafficking) (Fig. S11A and S11B). Using the afore mentioned degradome data from tomato plants infected with TYLCV [82], we found that at 21 dpi, 102 of the DEphasiRNAs could target the degradation of 120 tomato transcripts, and the inverse correlation between the expression of the deregulated phasiRNAs and their target genes was observed only in approximately 40% of the pairs (Fig. S12).

**Fig. 8:**
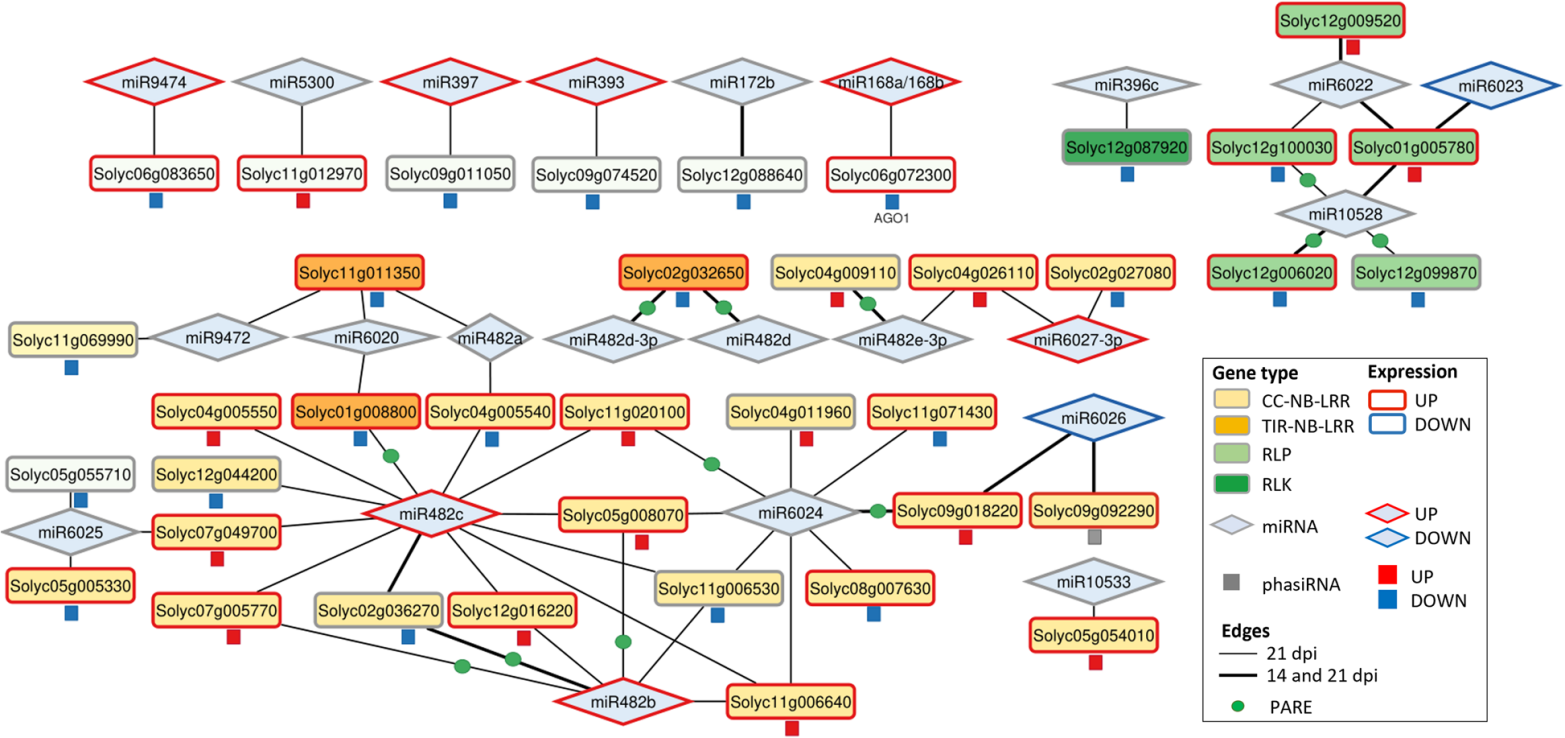
Tomato miRNA-*PHAS* loci-phasiRNA network upon TYLCV infection at 21 dpi. Network representation of the miRNAs (21 and 22-nt, rhombus) that trigger the formation of DEphasiRNAs (squares) from their target *PHAS* loci transcripts (rectangles) at 21 dpi. Each geometrical form is surrounded by a colored line that indicates their differential expression pattern: red for induced, blue for repressed and gray when they are not differentially expressed. The miRNA isoform that triggers each *PHAS* loci is indicated in Dataset S7. The different types of *PHAS* loci are marked by colors and indicated in the figure legend. The black edges connect a miRNA and a targeting *PHAS* locus, the thick ones indicate that the pair was also observed at 14 dpi (Fig. S13). miRNA-*PHAS* locus pairs that have also been identified using the degradome analysis are marked with a green circle

We selected the *PHAS* loci whose transcripts were putative target genes of 21- and 22-nt miRNAs and whose phasiRNAs were deregulated during infection. To integrate their interactions in a more visual and comprehensive manner, we built a network that showed the expression profiles of the initial miRNA triggers, the *PHAS* loci and the DEphasiRNAs at 14 dpi (Fig. S13) and 21 dpi (Fig. 8). The number of miRNA/*PHAS* loci interactions in the network increased at 21 dpi, although some of them were already established at 14 dpi (thick black lines in Fig. 8 and Fig. S13). The network showed that at 21 dpi, 24 miRNAs targeted 38 *PHAS* loci that were essentially plant immunity genes such as RLKs, RLPs and NLRs (CNLs and TNLs) (Fig. 8, Table 3). Among the miRNA controlling the production of *NLR*-derived phasiRNAs deregulated by TYLCV, we found members of the miR482/2118 conserved superfamily, as well as *Solanaceae* specific ones such as sly-miR6026 and sly-miR6027 [85–87]. The network highlighted a high level of interlinking produced by miRNAs that target many loci (e.g., sly-miR482 and sly-miR6024), and *PHAS* loci whose transcripts could be targeted by miRNAs from different families (Fig. 8). It is important to point out that some of the miRNA*-PHAS* loci interactions proposed in the network were also detected using the tomato degradome dataset from TYLCV-infected tomato plants [82] (green dots in Fig. 8 and Fig. S13).

**Table 3:** Tomato *PHAS* loci that produced DEphasiRNAs at 14 and 21 dpi and their miRNA triggers

*dpi*	*miRNA*	*PHAS loci*	*DEphasiRNA*
14	11	3 RLP 4 CNL 1 TNL 1 Misc	78
21	24	5 RLP 1 RLK 21 CNL 2 TNL 2 NBS-LRR 7 Misc	209

*RLP* Receptor-Like Proteins, *RLK* Receptor-Like Kinases, *CNL* Coiled-coil N-terminal Nucleotide-binding Leucine-rich repeat receptor, *TNL* Toll/interleukin-1 N-terminal Nucleotide-binding Leucine-rich repeat receptor, *NBS* Nucleotide Binding Site, *LRR* Leucine Rich Repeat, *Misc* Miscellaneous

### DNA methylation landscape of the tomato genome upon TYLCV infection

To evaluate the impact on the plant methylome during geminiviral infection, we performed whole genome bisulfite sequencing (WGBS) on two biological replicates from mock and TYLCV-infected tomato plants used to analyze the transcriptome and sRNA profile at 14 dpi (Dataset S1). On average, 132 million 100-bp paired-end reads per sample (ranging between, 128–137 million) were obtained, which in total contained more than 395 million uniquely mapped reads to the tomato genome, with an average coverage of 23.6 × and a mean conversion rate based on the cytosine methylation levels in the chloroplast genome for the four samples of 99.74% (Dataset S15). The WGBS reads from infected samples were also mapped to the TYLCV genome and the characterization of the virus methylome at 14 dpi, was previously described [55].

**Fig. 9:**
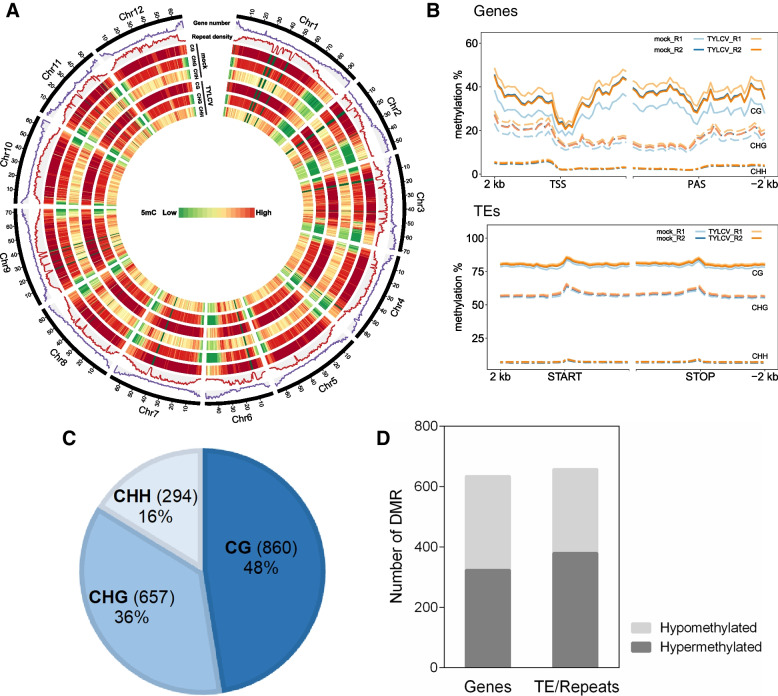
Tomato epigenome landscape upon TYLCV infection. **A** Density plot of 5-methylcytosine in different contexts (CG, CHG and CHH) across tomato chromosomes for TYLCV-infected and mock samples. Chromosomes names are indicated on the outer rims. **B** Methylation rate in genes and TEs/repeats in the two biological replicates (R1 and R2) from mock and infected plants. Transcriptional start site (TSS) and polyadenylation sites (PAS) are indicated. **C** Total number and % of DMRs(TYLCV/mock) on each methylation context (CG, CHG and CHH). **D** Number of hypo-methylated and hyper-methylated regions in genes and TEs/repeats

More than 60% of the tomato genome consists of heavily methylated transposable elements that are concentrated in the pericentromeric heterochromatin regions [1]. Distribution of the 5-methylcytosine levels in the three sequence contexts (CG, CHG, CHH, where H = A, C or T) across the tomato genome in mock and TYLCV-infected plants, revealed no significant genome-wide changes in DNA methylation levels upon the infection (Fig. 9A, Fig. S14). We could detect some differences between infected and mock plants, in the CG and CHG methylation levels at the TSS and PAS of genes but with some variation between biological replicates that did not allow us to arrive to a robust conclusion (Fig. 9B). Specific analysis of the methylation levels at genes and TEs/repeats for each methylation context, revealed that no substantial changes occurred at the promoters of genes or at TEs/repeats upon TYLCV infection (Fig. 9B). However, the expression of the main genes that control DNA methylation were generally slightly induced in TYLCV-infected plants (DNA methyltransferases, DNA demethylases, proteins involved in RNA-dependent DNA methylation (RdDM), and chromatin factors such as histone modifying enzymes and chromatin remodeling complexes) (Table 4).

**Table 4:**
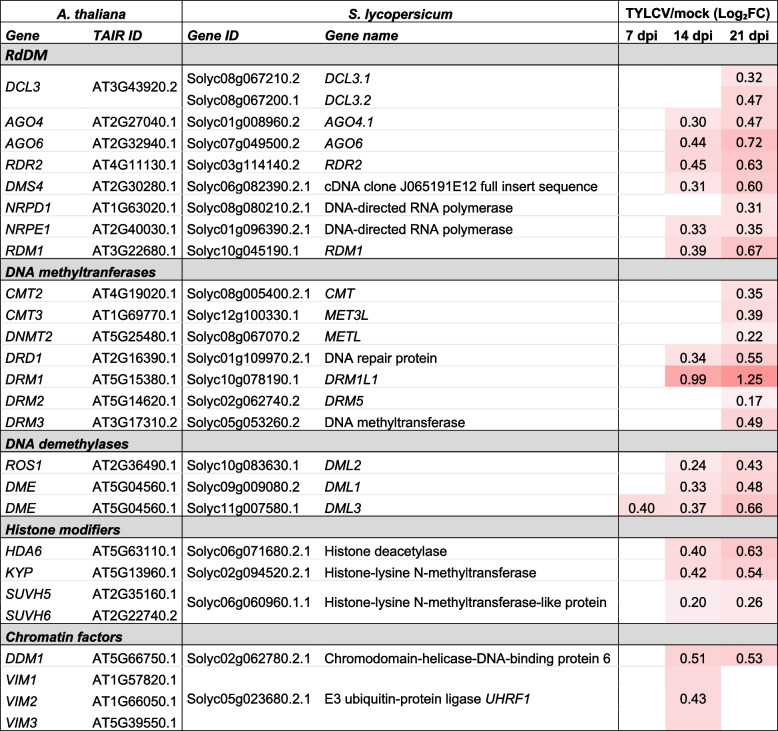
Differentially expressed tomato genes involved in transcriptional gene silencing during TYLCV infection

To identify regions in the tomato genome embracing changes in DNA methylation upon TYLCV infection, we determined the differentially methylated regions (DMRs). We found 1811 DMRs in the three DNA methylation contexts that were associated with hyper- or hypomethylation. The CG context showed the greatest amount of DMRs (860) and the CHH context the smallest (294) (Fig. 9C). The number of the DMRs was comparable in genes (635) and TEs/repeats (658), and the ratio between hyper- and hypomethylated DMRs was higher in genes (0.96) than in TEs/repeats (0.73) (Fig. 9D). As DNA methylation in plants could be established by RdDM, we identified the genomic regions to which tomato 24-nt siRNAs were mapped and determined changes in their accumulation upon TYLCV infection. No genome-wide correlation was found between the DMRs and the changes in the accumulation of siRNAs for the 24-nt siRNA enriched loci (data not shown, same result for 21- and 22-nt siRNA enriched loci).

DNA methylation is a common epigenetic mark that is associated with the inactivation of transcription and therefore changes in DNA methylation can influence gene expression [88, 89]. To integrate the information of the tomato transcriptome and methylome obtained during TYLCV infection, we checked whether there was correlation between the loci encompassing DMRs (hyper- or hypomethylated regions) and their expression level. A total of 635 of the DMRs were mapped in 597 genes, and 6.2% of them were differentially expressed (37 DEGs) (Table 5). Among the 16 hypermethylated genes, the percentage of induced (81%) versus repressed (19%) genes (Table 5) was very similar to the proportion of the entire induced and repressed genes during TYLCV infection (78% and 22%, respectively) (Fig. 1B). On the other hand, from the hypomethylated regions that overlapped with DEGs (21), 9% were downregulated and 91% (19 genes) were induced, suggesting that the reduced methylation at these genes could be regulating their induction. Interestingly, 4 from those 19 induced and hypomethylated genes were involved in defense response.

**Table 5:**
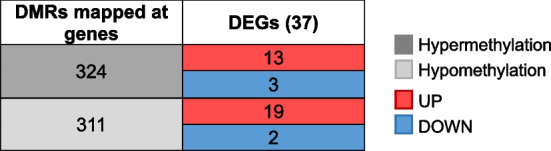
DMRs that mapped at genes in TYLCV-infected tomato plants at 14 dpi

## Discussion

In this study, we performed an integrative analysis to decipher plant gene regulation at different levels in response to the viral infection using a relevant agronomic system: tomato and the geminivirus TYLCV. To address this objective, we set up an experimental design to determine the changes in the mRNA and sRNA transcriptome at significant steps of the infection process: before the viral accumulation was detected (2 dpi), when the amount of viral molecules was exponentially growing but the plants were still symptomless or showed light symptoms (7 dpi), and when the infection was well stablished with the highest levels of viral DNA accumulation and severe symptoms in all plants (14 and 21 dpi). The study was completed with the characterization of the changes in the host methylome upon TYLCV infection at 14 dpi. To evaluate the biological significance of the results, we must contemplate that the data correspond to a systemic infection from tomato apical leaves, in which the vast majority of the cells analyzed do not contain the virus, since TYLCV just replicates and accumulates in phloem-companion cells (0.8% to 2% of the leaf cells, unpublished results) [90]. In this scenario, we must consider that the variations in host expression mainly corresponded to changes generated in infected and uninfected cells and therefore, it is likely that the specific alterations in the infected cells, go unnoticed. Omics approaches at a single cell resolution will be required to accurately characterize the molecular and physiological changes of plant-geminivirus interaction.

In plants with no or weak symptoms, we detected limited changes in the transcriptome, although the quantification of viral DNA by qPCR showed that TYLCV was actively replicating (7 dpi). From then on, the transcriptomic variations detected seem to depend on the long-lasting viral presence and not in the amount of viral DNA since they correlated with the increase in symptom intensity seen from 7 to 21 dpi but not with the viral titer.

Limited commonalities arise when comparing the functional categorization of tomato deregulated genes with the four transcriptomic analyses performed with TYLCV-related viruses (TYLCSV in *S. lycopersicum* [36, 38] or TYLCV in *S. lycopersicum* [40] or *N. benthamiana* [41]), probably due to the diverse experimental conditions on each study, including the sample collection time points (ranging from 14 to 56 dpi). Common overrepresented categories with at least one of the four mentioned studies, were observed for the induced genes (cellular response to stress, regulation of transcription, autophagy, intracellular transport, and abscisic acid metabolism), as well as for the repressed ones (terms related to photosynthesis and carbohydrate metabolic processes).

To obtain a comprehensive representation of the transcriptional changes, we took advantage of our experimental design and analyzed the transcriptional profile of the DEGs through time. Of particular interest were the two defense-related processes that were over-represented throughout the infection (7, 14, and 21 dpi) among the induced genes, RNAi and the immune response (Fig. 2, Fig. S4). Genes involved in RNAi-mediated antiviral defense, such as *DCL2*, *DCL4*, *RDR1* and *AGO2* were upregulated at the onset of infection (7 dpi) and showed the highest levels of induction at 21 dpi (Table 1). A similar behavior was observed for *AGO7* which is associated with the production of secondary siRNAs in Arabidopsis [15, 16]. Moreover, the expression level of the susceptible allele of the TYLCV-resistance gene, *Ty-1* (RDR3.2), was just slightly induced at the late stages of TYLCV infection, as previously described [91] (Table 1). Similarly, plant immune receptors such as NLRs or RLKs/RLPs, were induced particularly at the late stages of TYLCV infection (Fig. 2, Fig. S4). Among them, *Sw5a*, a CNL gene present in our tomato variety, (has been described as a resistance source against the begomovirus ToLCNDV [25]. In ToLCNDV-infected tomato plants, *Sw5a* showed sixfold upregulation and sevenfold downregulation in resistant and susceptible cultivars, respectively. However, in our susceptible variety, *Sw5a* was just weakly upregulated in TYLCV-infected plants (1.2-fold) at the same time of infection (21 dpi). *Sw5a* expression is controlled through the transcription factor SlMYB33 (*SlGAMYB1*, *Solyc01g009070*) whose accumulation depends on the action of the sly-miR159 [25]. Changes in the expression of this miRNA in resistant plants, lead to a rise in the accumulation of *SlMYB33* that produce an increase in *Sw5a* transcription. In susceptible plants, this miRNA is induced, which correlates with a reduction in the expression of the transcription factor and consequently of *Sw5a*. In contrast, in our TYLCV-infected susceptible plants, although we also observed a rise in sly-miR159 levels, *SlMYB33* accumulation was slightly increased, which could account for the observed *Sw5a* upregulation (Table 6). We also observed the upregulation of sly-miR319 which have been shown to also target *SlMYB33* [92]. The mechanism by which the presence of geminiviruses induces sly-miR159 accumulation in susceptible plants is not known, nor why its induction causes the repression of *SlMYB33* in plants infected with ToLCNDV but not in TYLCV-infected plants.

**Table 6:**
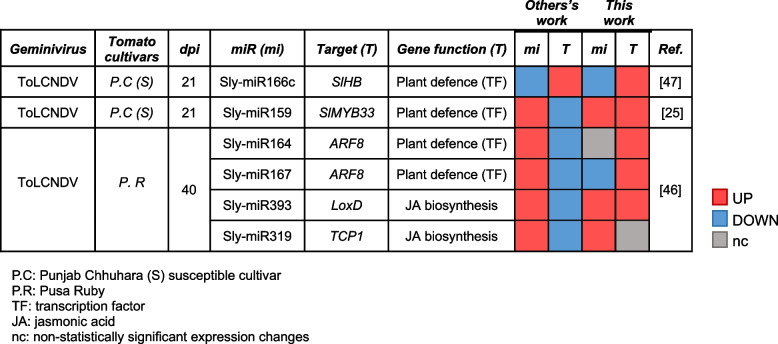
Differentially expressed miRNAs and their tragets involved in geminivirus-tomato interaction [47, 25, 46]

The categories comprising extracellular immune receptors (RLKs/RLPs) and some PTI downstream elements, such as RLCKs, were overrepresented among the genes upregulated during the infection, suggesting that induction of PTI receptors is part of the plant's defence response to the geminiviral infection. This result opens the possibility that, as occurs with NIK1 [31, 93], other RLKs/RLPs could participate in the defense against geminiviruses [31, 93].

During TYLCV infection, there was a substantial induction of many of the members of the gene silencing machinery that appeared to primarily target the production of virus-derived sRNAs (vsRNAs), that at 21 dpi represent more than 5% of the total sRNA reads [55]. However, this gene induction did not have a significant impact in the size or the distribution of the total population of tomato sRNAs. The number of 24-nt differentially expressed hetsiRNAs that mapped at genes and TEs/repeats, showed a significant increase from 14 to 21 dpi (around sixfold) and 245 of the hetsiRNA enriched genes were deregulated at 21 dpi, suggesting a transcriptional control of gene expression at the later stages of infection (Fig. S7 and Datasets S5-S10). Nevertheless, we could not find siRNA enriched loci that overlapped with DMRs at genes or TEs/repeats. On the other hand, the presence of the virus did induce changes in the miRNA-mediated gene regulation which was especially evident once the symptoms have developed. miRNAs are crucial regulators of the plant immunity, influencing various aspects of defense responses against pathogens by fine-tuning the expression of key genes involved in immune signaling such as genes encoding for ETI receptors (NLRs), transcription factors, and components of defense signaling pathways [94]. Throughout TYLCV infection, we identified 33 miRNA families whose expression was deregulated with a similar number of miRNAs induced and repressed at 21 dpi (Fig. 5). Although, as with mRNAs, the number of deregulated miRNAs increased with time during the infection, changes in miRNAs expression seemed to occur later than the deregulation of protein-coding genes (Fig. S15). These results suggested that the changes in the expression of the host miRNAs did not directly depend on the amount of viral DNA, viral transcripts or vsRNAs which maintained similar levels at 14 and 21 dpi (Fig. S2) [55]. Among the DEmiRNAs we found sly-miR6026, which targets the CLN resistant gene *Tm 2*^*2*^ as well as *DCL2*, which in turn is responsible for synthesizing the 22-nt sly-miR6026 [95, 96]. Reduction of sly-miR6026 expression using a target mimic RNA, increases *DCL2* expression and enhances resistance to potato virus X and TMV in tomato [96]. During TYLCV infection, sly-miR6026 was repressed, while the accumulation of *Tm 2*^*2*^ and *DCL2* was induced. Although this canonical inverse relationship between a miRNA and its target transcript correlated with that expected for a miRNA-mediated control of gene expression, the non-canonical pattern was widely observed when the general overview of the miRNA-target pairs was considered (Fig. 6). There are several possible explanations for this observation. First, considering that the analysis has been performed using a “in silico” prediction of miRNA-target pairs, the inverse relation between the levels of expression of the miRNA and its “real” target gene could be masked. However, this lack of correlation was also observed when the published degradome for TYLCV-infected tomato plants [82] was used to predict the target genes in our infected plants (Fig. S10). Second, we should consider that the miRNA and their putative target transcripts could be expressed in different tissues/cells and therefore, no correlation in the expression of both is expected. Third, the transcription factor-mediated induction of the target gene could hide the regulatory effect of a certain miRNA. Finally, we could not rule out the possibility that as it has been described in Arabidopsis, moss and rice, some miRNAs could be transcriptionally controlling the expression of their target [97, 98].

Three studies using massive sequencing approaches have identified tomato miRNAs and their target genes deregulated during ToLCNDV infection [25, 46, 47]. Although we found that some of these miRNA-target pairs were also deregulated upon TYLCV infection, the trend of the changes, induction/repression, were in all cases, except for sly-miRNA166c-*SlyHomeoBox* (*SlyHB*), different to the ones previously described (Table 6). These observations suggested that the deregulation of the expression of the miRNA-target pairs was dependent on the host-geminivirus interaction.

Although the deregulation of mRNA and miRNA during geminivirus infection has been documented, there is no data on the impact of geminiviral infection on phasiRNA accumulation. Previous studies have shown that plant sensing of a pathogen causes the downregulation of the miRNAs that control the production of phasiRNAs from *NLR* genes and that changes in the levels of these miRNAs, alter resistance against virulent pathogens, including viruses [95, 99–101]. How miRNA/NLR/phasiRNA regulation impacts the pathogen-host interaction is not clearly understood. Two hypotheses have been proposed: (i) control of NLRs by miRNAs/phasiRNAs could serve as a link between the pattern recognition receptors and NLR-mediated responses, increasing the availability of NLRs when a pathogen is detected; (ii) considering the detrimental effects of NLR expression, miRNA/phasiRNA downregulation of NLRs could function as a feed-back system to reduce the potential fitness losses when the pathogen is no longer a danger [87, 100]. During TYLCV infection we detected an increase in the number of DEphasiRNAs over time (Fig. 7) which followed a similar dynamic to the DEmiRNAs (Fig. S15). Overall, there was a generalized induction in the accumulation of DEphasiRNAs derived from NLRs and RLKs at 21 dpi, suggesting that the transcripts of these genes were being cleaved by their miRNAs. Most DEphasiRNAs derived from NLRs were triggered by three miRNAs: miR482b, miR482c and miR6024 (which triggered among other CNLs, *Tm 2*^*2*^). However, when considering the relationship between the expression of the miRNAs, *PHAS* loci and phasiRNA accumulation upon TYLCV infection, a complex scenario emerged. The relation in the level of expression of this triplet, was very heterogeneous, even if only miRNA-target interactions confirmed by experimental results or degradome analysis were considered. For example, the expression of sly-miR482c/482b and their target-derived phasiRNAs were mostly induced, while the expression of the *PHAS* loci could be induced or repressed (Fig. 8). Similar discrepancies in the expression among sly-miR482/2118 and its *NLR* targets have been described in tomato plants, in spite that the reduction in the miRNA accumulation confers resistance to bacterial and oomycete pathogens [101].

The control of gene expression mediated by miRNA/phasiRNA also affect other relevant loci such as *AGO1* which encodes an RNA slicer that selectively recruits miRNAs and siRNAs, and is targeted for degradation by silencing suppressor F-box-containing proteins from RNA viruses [102, 103]. Transcripts targeted by an AGO1/22-nt sRNA complex can attract components of the PTGS amplification machinery allowing their transformation into dsRNAs, leading to the production of secondary siRNAs and their subsequent loading onto AGO1 proteins, which maximizes the elimination of viral RNAs from the plant cell [84]. Upon TYLCV infection, the expression of both sly-miRNA168 and its target AGO1, were induced, while the AGO1-derived phasiRNAs were reduced. This result suggested that despite of the coexpression of AGO1 and miRNA168, previously described in Arabidopsis [104], the posttranscriptional control of sly-miRNA168 on AGO1 was impaired during TYLCV infection.

To the best of our knowledge, this study represents the first analysis of the changes in the tomato methylome during a geminivirus infection. Tomato genome has approximately 900-megabase (Mb) and genome-wide DNA methylation analyses have revealed that it is extensively methylated and more than 60% of its genome consists of methylated repeats and transposable elements [1, 71]. In tomato leaves, the overall methylation level is around 22% and CG and CHG methylation shows the highest level (85.51% and 56.15%, respectively) while methylation in the CHH context is the lowest one (8.63%) [71]. In our study, there were no significant genome-wide changes in the methylome of TYLCV-infected tomato plants at 14 dpi (Fig. 9A, Fig. S14). Although an increase in DNA methylation could be detected at the promoters and polyadenylation sites of genes when the infected and mock samples were compared, additional replicates will be needed to statistically confirm this difference (Fig. 9B). On the other hand, we could detect DMRs for a small percentage of genes and TEs/repeats and almost half of the changes upon TYLCV infection occurred at the CG context suggesting that they were not directly dependent on 24-nt siRNAs. In fact, we did not find changes in the accumulation of siRNAs for the 24-nt hetsiRNAs (or 21- and 22-nt) enriched loci that overlapped with DMRs at genes or TEs/repeats. We detected a slight but general induction of most of the main players in maintaining or establishing DNA methylation which could be responsible for the changes in DNA methylation levels at certain loci (Table 4). Previous data showed that geminivirus infection repressed the expression of the maintenance DNA methyltransferases, *NbMET1* and *NbCMT3*, in systemic infection in *N. benthamiana* [50] but this did not seem to be the case in tomato.

DNA methylation at cytosines is one of the several epigenetic mechanisms that eukaryotic cells use to control gene expression and transcriptionally silenced regions are typically hypermethylated. We took advantage of having the transcriptome and methylome data from the same samples and looked for DEGs whose DNA methylation levels have changed upon TYLCV infection. Our data indicated that the majority of the genes that were hypomethylated, were also upregulated, suggesting that this epigenetic mark could be controlling the expression of at least some of these induced genes. Further work will be needed to determine the biological relevance of these findings.

Although we cannot rule out the possibility that the effect of geminivirus suppressors on the host methylome could be just restricted to certain loci, we also have to consider that the impact of TYLCV infection on the tomato methylome could be masked due to the dilution effect that represents to determine the changes in the host methylome using a whole leaf in a phloem-limited virus. Additionally, considering that we have analyzed the plant methylome at 14 dpi, it could be possible that the changes will be greater at later time points, after a longer exposure to the virus.

Besides DNA methylation, we cannot discard a possible effect of geminiviruses on the host epigenome based in the alteration of other epigenetic marks different to DNA methylation, such as histone modifications or nucleosome composition. At least another epigenetic mark related to gene silencing such as methylation of histone H3 at lysine 9 (H3K9me), has been involved in the response to geminivirus infection [51]. Other approaches such as chromatin immunoprecipitation followed by massive sequencing (ChIP-seq) should be performed in infected plants to further characterize the relevance of these marks on the host epigenome during viral infection. TYLCV, is seed-borne but not seed-transmitted and has been detected in the reproductive tissues of *N. benthamiana* and tomato [105]. If the viral presence in those tissues can induce epigenetic changes that could be transgenerationally inherited, constitutes a tantalizing hypothesis that needs further investigation.

The overall results from our study showed that TYLCV infection induced changes in tomato plants at transcriptional and post-transcriptional levels, inducing among others, gene silencing and the plant immunity machinery. In this situation, the main question is how the virus, despite the enormous display of defense systems, can complete its infection cycle. Among other more complex ones, the outcome of the virus-plant interaction could be explained by two scenarios: (i) the differential timing between the establishment of the defense systems and the virus replication and movement, disabling the temporal deployment of control measures required to efficiently prevent viral infection (ii) the generation of counter defense measures by the virus such as the expression of silencing suppressors and the interference with the translation signal generated by the plant's immune system.

## Conclusions

Our results show that TYLCV induces substantial transcriptional changes in tomato that increase throughout the infection and that are dependent on the amount of viral DNA at the initial stages but not once the viral titer has reached its maximum level. Genes that belong to the two main defense mechanisms in plants, gene silencing and the immune response, are induced before the symptoms are established. The induction of those genes increases in intensity and/or in number throughout the infection. On the other hand, the deregulation of tomato miRNAs and phasiRNAs do not rely on the amount of viral DNA, viral transcripts or viral sRNAs and their significant induction or repression appear after the substantial deregulation of protein-coding genes. The analysis of the differentially expressed miRNAs, showed that they mainly target genes involved in auxin response, gene silencing, or genes encoding transcription factors and immune receptors (RLKs and NLRs), many of which are *PHAS* loci that produce deregulated phasiRNAs. Interestingly, the expected inverse relationship between a miRNA and its target was not consistent when the general overview of the miRNA-target pairs was considered. The expression of most of the main genes from the DNA methylation machinery are slightly induced during TYLCV infection and have identified differentially methylated regions that could be involved in the transcriptional regulation of some of the differentially expressed genes. Taken together, this study provides insights into the complex interaction of TYLCV and tomato interaction and represents the first integrative and comprehensive analysis of the changes in the tomato mRNA transcriptome, sRNA profile, and methylome, during a geminivirus infection.

## Methods

### Plant growth and viral inoculation

Tomato plants (Solanum lycopersicum cv. Moneymaker) were grown as indicated in [55] and agroinfection was performed as described in [106]. Briefly, *Agrobacterium tumefaciens* LBA4404 strain was used to infect three-week-old tomato plants with a clone of the TYLCV isolate [ES:Alm:Pep:99] (AC: AJ489258) [106] by infiltrating the axillary bud between the third and fourth tomato leaves. As controls, we used mock–inoculated plants infiltrated with Agrobacterium carrying the binary vector and naïve non-inoculated plants.

### Sample collection, nucleic acid extraction and relative viral DNA quantification

Sampling, DNA and RNA extractions and relative viral DNA quantification were performed as described in Piedra-Aguilera et al., [55]. Symptoms development was assessed according to the following scale: 0-No symptoms, 1-Slight yellowing (very mild symptoms); 2-Slight leaf curling and more yellowing (mild symptoms); 3- Strong yellowing, curling and slight cupping (moderate symptoms); 4- Stunting and strong curling and cupping; (severe symptoms); 5- Severe stunting, inhibition of plant growth (very severe symptoms) (Fig. S2).

### Libraries construction and sequencing

RNA-seq, sRNA-seq and WGBS libraries from naïve, mock and TYLCV-infected plants were generated as described in Piedra-Aguilera et al., [55].

### RNA-seq data analysis

RNA-seq paired-end reads were mapped against the *S. lycopersicum* reference genome (SL2.5) using STAR version 2.5.1b [107] with ENCODE parameters for long RNA. Genes were quantified using RSEM version 1.2.28 [108] with default parameters and the ensembl release 31 annotation. Differential expression analysis was performed in R using the limma package [109]. Lowly expressed genes were filtered by retaining only genes with normalized read counts above 50 in at least three samples, followed by voom transformation, linear model fit, and statistics calculation for defined contrasts using the eBayes function. Expression heatmaps were drawn by calculating sample Euclidian distances between normalized counts and plotted using R package heatmap. The differentially expressed genes (DEGs) were determined by comparing TYLCV-infected samples versus mock samples (ratio ≥ 1.5-fold or ratio ≤ 0.75-fold) with FDR adjusted *p*-values ≤ 0.05.

### sRNA-Seq data analysis

The raw sRNA sequencing data were firstly preprocessed to remove adapter sequences, low-quality and low complexity reads, and reads shorter than 18-nt and longer than 26-nt using cutadapt [110] and Filter Tool of the UEA small RNA Workbench [111]. Subsequently, sRNA reads were additionally filtered to exclude reads matching to rRNAs, tRNAs, snRNAs, snoRNAs in RNACentral database [112]. To identify known tomato miRNAs, remaining preprocessed sRNA reads were compared to tomato miRNAs registered in the miRBase database release 22 allowing no mismatches [113]. To identify novel unannotated miRNAs and their loci of origin (*MIR* loci), reads were submitted to the two plant miRNA prediction tools ShortStack [114] and miR-PREFeR [115]. Predictions were performed using default parameters, except that no mismatches were allowed during mapping on reference *Solanum lycopersicum* genome v2.5. Novel miRNAs were identified if they had more than five raw reads in at least two sRNA libraries, and their sequences, and corresponding miRNA* and *MIR* loci were predicted with both miRNA prediction tools. Within prediction analysis, the reads that were mapped to more than 30 locations in the tomato genome were also discarded. The output of miRNA prediction tools also contained the predictions of already annotated tomato *MIR* loci, therefore to separate them from potential novel *MIR* loci candidates, annotated tomato pre-miRNA precursors from miRBase database release 22 were mapped to reference tomato genome using bowtie2 [116]. Next, genome locations were extracted and compared with predicted *MIR* loci locations using our internally developed script [117]. If no overlap was detected, the predicted *MIR* loci were regarded as novel *MIR* loci. Novel tomato miRNAs were further classified into known or novel miRNA families by clustering their predicted pre-miRNA sequences with sequences of known plant pre-miRNAs from miRBase using CD-HIT-EST with an identity threshold of 0.8 [118]. Sequences showing similarities with annotated pre-miRNAs were grouped into corresponding known miRNA families, and sequences that did not show similarity with known plant miRNAs were classified as novel miRNA families. Additionally, miRNA variants (isomiRs) of known and novel miRNAs were identified using computational pipeline isomiRID [119]. Only sRNAs perfectly matching to known or novel tomato pre-miRNA sequences, known as templated isomiRs, were considered. Prediction of *PHAS* loci and phasiRNAs was performed using unitas tool [120]. *PHAS* loci and phasiRNAs were detected by mapping preprocessed sRNA reads to tomato ITAG v2.4. Analysis of phasing was performed in 21- and 24-nt intervals using default settings [121]*.*

### sRNA quantification and statistical analysis

Preprocessed reads from sRNA-seq samples were mapped with no mismatches to all identified known, and novel miRNAs, miRNA variants (isomiRs), and phasiRNAs using bowtie2 [116] and based on the alignments the abundances of miRNAs (variants) and phasiRNAs were counted using a custom script that was previously published by Križnik et al. [117]. Differential expression analysis of miRNAs and phasiRNAs between TYLCV-infected and mock samples was performed using the limma package in R [122]. Briefly, sRNA counts with a baseline expression level of at least 50 reads in at least two of the same biological replicate samples were TMM-normalized using edgeR package [123] and analyzed using the voom function [109]. To identify differentially expressed miRNA and phasiRNAs, the empirical Bayes approach was used, and the resulting p-values were adjusted using Benjamini and Hochberg’s (FDR) method. Adjusted *p*-values ≤ 0.05 were considered statistically significant. Just DEmiRNAs with at least 10 CPM (counts per million) in any of the samples in both biological replicates and FDR adjusted p-value ≤ 0.05, were considered for further analysis.

To identify differentially expressed siRNA loci (DEsiRNAs), cleaned sRNA reads were mapped to tomato genome with ShortStack [114], which also reported the raw counts for each siRNA loci based on read alignments. The raw counts were then fed to DESeq2 [124] to identify DEsiRNAs between TYLCV-infected and mock samples, with a cutoff of adjusted *p*-values < 0.05. Genomic features including gene promoters, gene bodies and TEs/repeats overlapping with DEsiRNAs were defined with BEDTools [125].

### sRNA target prediction

In silico identification of tomato transcripts targeted by sRNAs was carried out using psRNATarget [78] and ITAG v2.4 tomato transcriptome sequences with default parameters, except the maximum expectation parameter was set to 3.0. Results of miRNA-target (*PHAS* loci) interactions were used to reveal miRNA triggers of the phasiRNA [126, 127]. The miRNA-*PHAS* locus-phasiRNA network was generated using Cytoscape [128] after selecting the *PHAS* loci that generated differentially expressed phasiRNAs (DEphasiRNAs) and identifying the miRNAs that triggered those *PHAS* loci according to psRNATarget.

### Degradome-seq target prediction

Four degradome datasets (GEO accession No. GSM1213988, GSM1213989, GSM1213990, GSM1213991) produced from TYLCV-infected and mock tomato leaves [82] were retrieved from the NCBI Gene Expression Omnibus database and analyzed with CleaveLand4 [129] using tomato sRNA sequences and the tomato transcriptome sequences (ITAG release 2.4). All identified degradation targets were classified into five categories as previously described [129]. Category “0” is defined as > 1 raw read at the position, with abundance at a position equal to the maximum on the transcript, and with only one maximum on the transcript. Category “I” is described as > 1 raw read at the position, with abundance at the position equal to the maximum on the transcript, and more than one maximum position on the transcript. Category “II” includes > 1 raw read at the position and abundance at the position less than the maximum but higher than the median for the transcript. Category “III” comprised the transcripts with > 1 raw read at the position, and abundance at the position equal to or less than the median for the transcript. Category “IV” comprised transcripts with one raw read at the cleavage position. Only categories with high confidence of cleavage (0, I, II, III) and p-value ≤ 0.05, were considered for biological interpretation and visual representation. Results of miRNA-target (*PHAS* loci) interactions were also used to confirm miRNA triggers of the phasiRNA production determined in silico.

### WGBS analysis

To remove potential PCR duplicates, read pairs having identical bases at positions of 10 to 80 in both left and right reads were defined as duplicated pairs and then collapsed into unique read pairs. The resulting reads were further processed to remove adaptor and low-quality sequences using Trimmomatic [130]. The trimmed reads were then mapped to the tomato genome using a methylation-aware aligner Bismark v0.17.0 (–bowtie1 -n1) [131]. The methylation information was extracted from the alignments by a script “bismark_methylation_extractor” provided in Bismark and the resulted cytosine reports were separated according to the cytosine context, CG, CHG and CHH. These cytosine reports were analyzed by R package methylKit v1.1.6 [132] to identify differentially methylated regions (DMRs) using a sliding window approach (window size = 100 bp and step-size = 50 bp). Methylation information was summarized in each window. Sites with too low (< 4x) or too high coverage (> 99.9th percentile of coverage in each library) were excluded from the analysis. CG and CHG windows with less than 4 cytosine sites and CHH windows containing less than 10 cytosine sites were also excluded. DMR were finally determined using logistic regression and were adjusted with the SLIM method implemented in methylKit [133]. Windows with methylation difference ≥ 25% between mock and TYLCV-infected samples and FDR adjusted *p*-value ≤ 0.01 were selected as DMRs and adjacent DMRs were further merged if they overlapped. Visualization of tomato genome-wide methylation levels in TYLCV-infected and mock samples at 14 dpi (Fig. 9) was generated using Circos [134].

### Gene enrichment analysis

Functional enrichment analysis by Gene Set Enrichment Analysis (GSEA) [56] was performed using non-filtered normalized read counts to search for regulated processes and functionally related gene groups, altered significantly by the viral infection (FDR adjusted *p*-value ≤ 0.05) using the tomato GoMapMan gene sets defined in the file “sly_SL2.40_ITAG2.3_2017-03–14.gmt” [57]. The analysis of the functional enrichment in biological processes from selected DEGs and miRNA or phasiRNA target genes was conducted by MapMan software [135, 136] and the GO enrichment analysis from the PANTHER classification system [137, 138]. For the Mapman enrichment analysis, we used the gene expression levels and gene sets based on the tomato GoMapMan ontology defined in the file “sly_SL2.40_ITAG2.3_2017-03–14.gmt” [57]. Ontology terms with Wilcoxon test and Benjamini–Hochberg FDR adjusted *p*-values ≤ 0.05 were considered significantly enriched. The functional enrichment analysis from the DEGs at 14 and 21 dpi was also represented for the immunity-related categories based on the pathway representation performed by Mapman (Fig. S4). For the GO enrichment analysis, we performed the PANTHER GO-Slim biological process analysis and the statistical overrepresentation test, biological process with FDR adjusted p-values ≤ 0.05 were considered significantly differentially enriched.

### Clustering analysis of the differentially expressed genes

For clustering analysis, the list of a total of 6301 unique genes differentially expressed in at least one comparison was further filtered using R [139] package maSigPro (version 1.72.0.) with significance level of 0.01 and cut-off level at the R-squared value of 0.8, resulting in 1770 transcripts. Clustering of these 1770 transcripts (log_2_FC values across the four different time points) was conducted using SplineCluster [Nick Heard, Gaussian process clustering of multidimensional time series, https://www.ma.imperial.ac.uk/~naheard/software/splinecluster/index.html]. Transcript profile figures and the heatmap figure were plotted using R package pheatmap (version 1.0.12.) [Raivo Kolde (2019). pheatmap: Pretty Heatmaps. https://CRAN.R-project.org/package=pheatmap].

### Supplementary Information

**Additional file 1. Fig. S1.** Experimental design for the transcriptome, sRNAome and methylome of tomato infected with the geminivirus TYLCV.

**Additional file 2. Fig. S2.** TYLCV accumulation and symptoms development in tomato plants during the infection.

**Additional file 3. Fig. S3.** Functional enrichment of upregulated genes upon TYLCV infection at 21 dpi.

**Additional file 4. Fig. S4.** Immunity-related categories (MapMan ontology) enriched in upregulated genes in response to TYLCV infection at 14 and 21 dpi.

**Additional file 5. Fig. S5.** Expression profiles of the tomato gene clusters during TYLCV infection.

**Additional file 6. Fig. S6.** Functional enrichment of the tomato gene clusters.

**Additional file 7. Fig. S7.** Differentially expressed tomato siRNA loci (DEsiRNAs) overlapped with gene promoters, gene bodies and TEs/repeats during TYLCV infection.

**Additional file 8. Fig. S8.** Tomato miRNA families.

**Additional file 9. Fig. S9.** Functional enrichment of the predicted target genes from the DEmiRNAs in response to TYLCV infection at 21 dpi.

**Additional file 10. Fig. S10.** Expression levels of the DEmiRNAs and their predicted target genes in tomato according to the degradome analysis.

**Additional file 11. Fig. S11.** Functional enrichment of putative targets of the DEphasiRNAs upon TYLCV infection at 21 dpi.

**Additional file 12. Fig. S12.** Expression levels of the DE phasiRNAs and their predicted target genes in tomato according to the degradome analysis.

**Additional file 13. Fig. S13.** Tomato miRNA-PHAS loci-phasiRNA network upon TYLCV infection at 14 dpi.

**Additional file 14. Fig. S14.** Percentage of DNA methylation at the three cytosine contexts (CG, CHG, CHH).

**Additional file 15. Fig. S15.** Dynamic of the deregulated tomato mRNAs, miRNAs and phasiRNAs during TYLCV infection.

**Additional file 16. Datasets. Dataset S1.** Total reads from RNAseq, sRNAseq and WGBS-seq. **Dataset S2.** Gene Set Enrichment Analysis (GSEA) of the differentially expressed genes. **Dataset S3.** Clustering of the differentially expressed genes. **Dataset S4.** sRNA-seq statistics. **Dataset S5.** Differentially expressed tomato siRNA loci (21-, 22- and 24-nt) overlapped with genes at 7 dpi. **Dataset S6.** Differentially expressed tomato siRNA loci (21-, 22- and 24-nt) overlapped with genes at 14 dpi. **Dataset S7.** Differentially expressed tomato siRNA loci (21-, 22- and 24-nt) overlapped with genes at 21 dpi. **Dataset S8.** Differentially expressed tomato siRNA loci (21-, 22- and 24-nt) overlapped with TEs/repeats at 7 dpi. **Dataset S9.** Differentially expressed tomato siRNA loci (21-, 22- and 24-nt) overlapped with TEs/repeats at 14 dpi. **Dataset S10.** Differentially expressed tomato siRNA loci (21-, 22- and 24-nt) overlapped with TEs/repeats at 21 dpi. **Dataset S11.** miRNAs expressed in tomato during TYLCV infection. **Dataset S12.** Degradome-seq analysis in tomato during TYLCV infection. **Dataset S13.** Predicted target genes for the sRNAs. **Dataset S14.** Differentially expressed phasiRNAs in tomato during TYLCV infection. **Dataset S15.** WGBS statistics.

## Data Availability

All data generated or analysed during this study are included in this published article (and its supplementary information files). Raw sequencing reads have been deposited in the NCBI BioProject database under the accession number PRJNA494932 (https://www.ncbi.nlm.nih.gov/bioproject/PRJNA494932). The datasets supporting the conclusions of this article are included within the article (and its additional files).
