# Supplementary material for: Transcriptional and epigenetic changes during tomato yellow leaf curl virus infection in tomato
Source: BMC Plant Biol. 2023 Dec 18;23:651. doi: 10.1186/s12870-023-04534-y (PMC10726652; doi:10.1186/s12870-023-04534-y)
Supplement: Supplementary file 1 — Additional file 1. Fig. S1. Experimental design for the transcriptome, sRNAome and methylome of tomato infected with the geminivirus TYLCV. [file 12870_2023_4534_MOESM1_ESM.pdf]

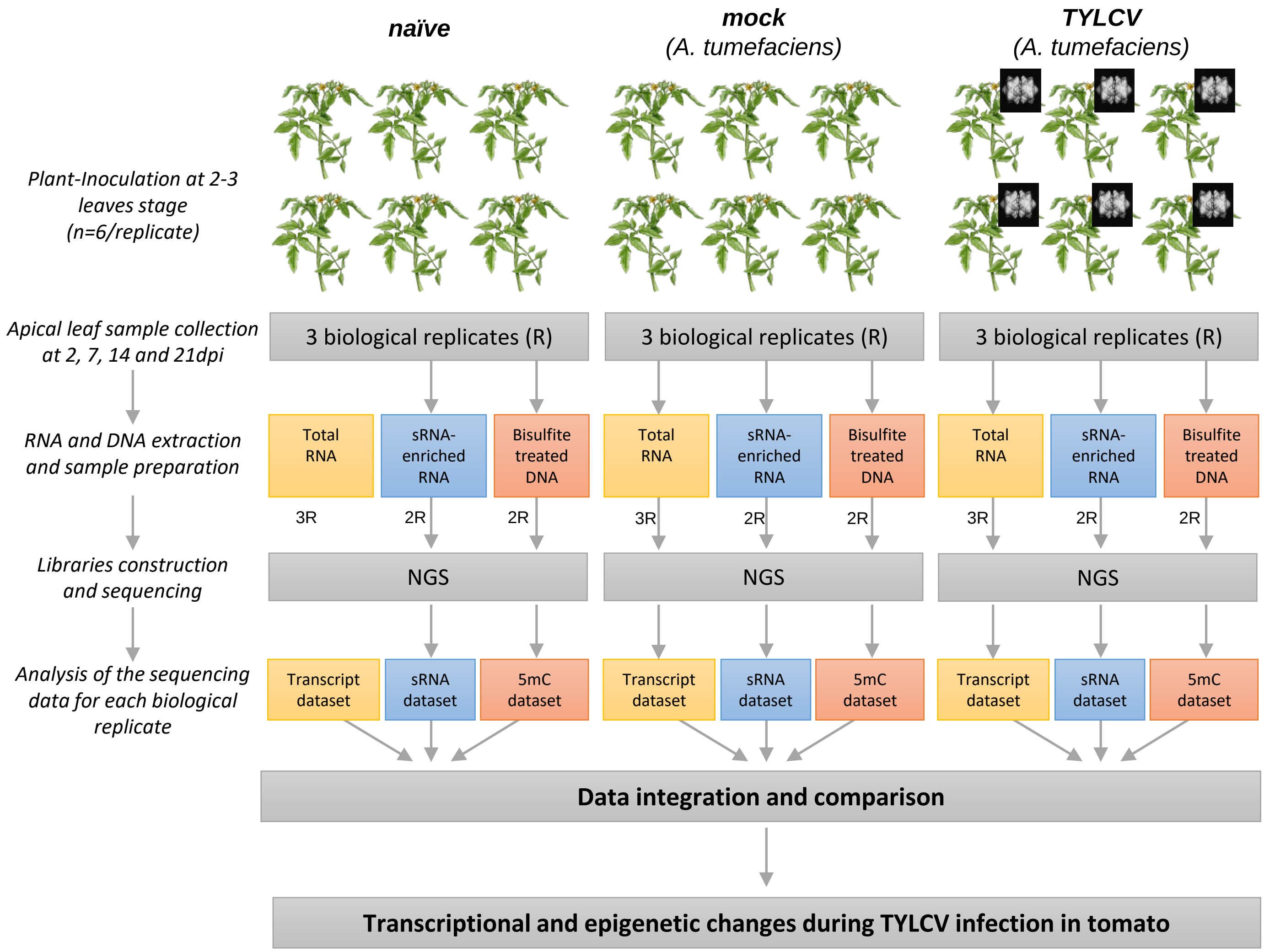

Additional file 1: Fig. S1. Experimental design for the transcriptome, sRNAome and methylome of tomato infected with the geminivirus TYLCV. Three-weeks-old tomato (*Solanum lycopersicum* cv. Moneymaker) plants were inoculated with *A. tumefaciens* carrying a clone of the isolate [ES:Alm:Pep:99] of *Tomato yellow leaf curl virus* (TYLCV) (AC: AJ489258) [135] As negative control, plants were mock inoculated with *Agrobacterium* carrying the empty vector and other plants remained non-inoculated as naïve plants. The second most recently expanded leaf from the apex was harvested at 4 different time points, 2, 7, 14 and 21 dpi. Three biological replicates of six separate infected plants' leaf tissue were pooled and used in downstream analysis for each time point. Total RNA and DNA was extracted from the three biological replicates and samples were prepared to analyze by NGS (next-generation sequencing) the transcriptome (3 biological replicates), smallRNAome and methylome (2 biological replicates each). The libraries were generated and sequenced and after the analysis of the sequencing data for each biological replicate the data was integrated and compared to determine the transcriptional and epigenetic changes during TYLCV infection in tomato.
