# Supplementary material for: Transcriptional and epigenetic changes during tomato yellow leaf curl virus infection in tomato
Source: BMC Plant Biol. 2023 Dec 18;23:651. doi: 10.1186/s12870-023-04534-y (PMC10726652; doi:10.1186/s12870-023-04534-y)
Supplement: Supplementary file 2 — Additional file 2. Fig. S2. TYLCV accumulation and symptoms development in tomato plants during the infection. [file 12870_2023_4534_MOESM2_ESM.pdf]

A

	Symptoms	
dpi	***mock***	***TYLCV***
2	0	0
7	0	1,5-2
10	0	3
14	0	4
21	0	5

B

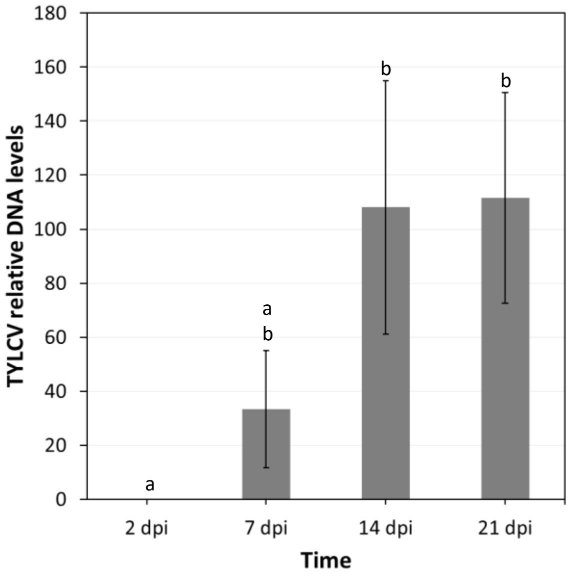

Additional file 2: Fig. S2. TYLCV accumulation and symptoms development in tomato plants during the infection. A) Symptoms development in control and TYLCV infected plants at 2, 7, 14 and 21 dpi. Symptoms scale: 0-No symptoms, 1-Slight yellowing (very mild symptoms); 2-Slight leaf curling and more yellowing (mild symptoms); 3- Strong yellowing, curling and slight cupping (moderate symptoms); 4-Stunting and strong curling and cupping; (severe symptoms); 5- Severe stunting, plant growth inhibition (very severe symptoms). B) Relative accumulation of TYLCV DNA in infected plants at 2, 7, 14 and 21 dpi. One-way ANOVA comparisons (Tukey's multiple comparisons test) shows statistically significant difference between the time points labelled with “a” and “b” (alpha 0.05).
