# Supplementary material for: Transcriptional and epigenetic changes during tomato yellow leaf curl virus infection in tomato
Source: BMC Plant Biol. 2023 Dec 18;23:651. doi: 10.1186/s12870-023-04534-y (PMC10726652; doi:10.1186/s12870-023-04534-y)
Supplement: Supplementary file 3 — Additional file 3. Fig. S3. Functional enrichment of upregulated genes upon TYLCV infection at 21 dpi. [file 12870_2023_4534_MOESM3_ESM.pdf]

Upregulated genes at 21 dpi (TYLCV/mock) *PANTHER GO. Slim Biological Process*	*GO terms*	*Ref-list (34652)*	*UP genes*	*Expect.*	*Fold enrich.*	*raw P-value*	*FDR*
Modification-dependent protein catabolic process	GO:0019941	308	65	37.6	1.73	1.51E-04	2.60E-03
Ubiquitin-dependent protein catabolic process	GO:0006511	291	61	35.52	1.72	2.74E-04	3.99E-03
Ubiquitin-dependent protein catabolic process via the multivesicular body sorting pathway	GO:0043162	11	9	1.34	6.7	1.16E-04	2.15E-03
Vesicle-mediated transport	GO:0016192	358	84	43.7	1.92	4.40E-07	1.52E-05
Multivesicular body sorting pathway	GO:0071985	27	14	3.3	4.25	6.44E-05	1.39E-03
Process utilizing autophagic mechanism	GO:0061919	45	14	5.49	2.55	4.83E-03	3.64E-02
Autophagy	GO:0006914	45	14	5.49	2.55	4.83E-03	3.66E-02
Autophagy of mitochondrion	GO:0000422	14	9	1.71	5.27	4.15E-04	5.32E-03
Autophagosome assembly	GO:0000045	23	10	2.81	3.56	2.01E-03	1.84E-02

Additional file 3: Fig. S3. Functional enrichment of upregulated genes upon TYLCV infection at 21 dpi. Gene ontology (GO) analysis of upregulated genes at 21 dpi (4980 genes with a ratio ≥1.5-fold, FDR adjusted p-value ≤0.05, when comparing TYLCV-infected samples versus mock samples) using the PANTHER GO-Slim biological process (statistical overrepresentation test). Only selected statistically significant biological processes are shown (FDR adjusted p-value p≤ 0.05).
