# Supplementary material for: Transcriptional and epigenetic changes during tomato yellow leaf curl virus infection in tomato
Source: BMC Plant Biol. 2023 Dec 18;23:651. doi: 10.1186/s12870-023-04534-y (PMC10726652; doi:10.1186/s12870-023-04534-y)
Supplement: Supplementary file 4 — Additional file 4. Fig. S4. Immunity-related categories (MapMan ontology) enriched in upregulated genes in response to TYLCV infection at 14 and 21 dpi. [file 12870_2023_4534_MOESM4_ESM.pdf]

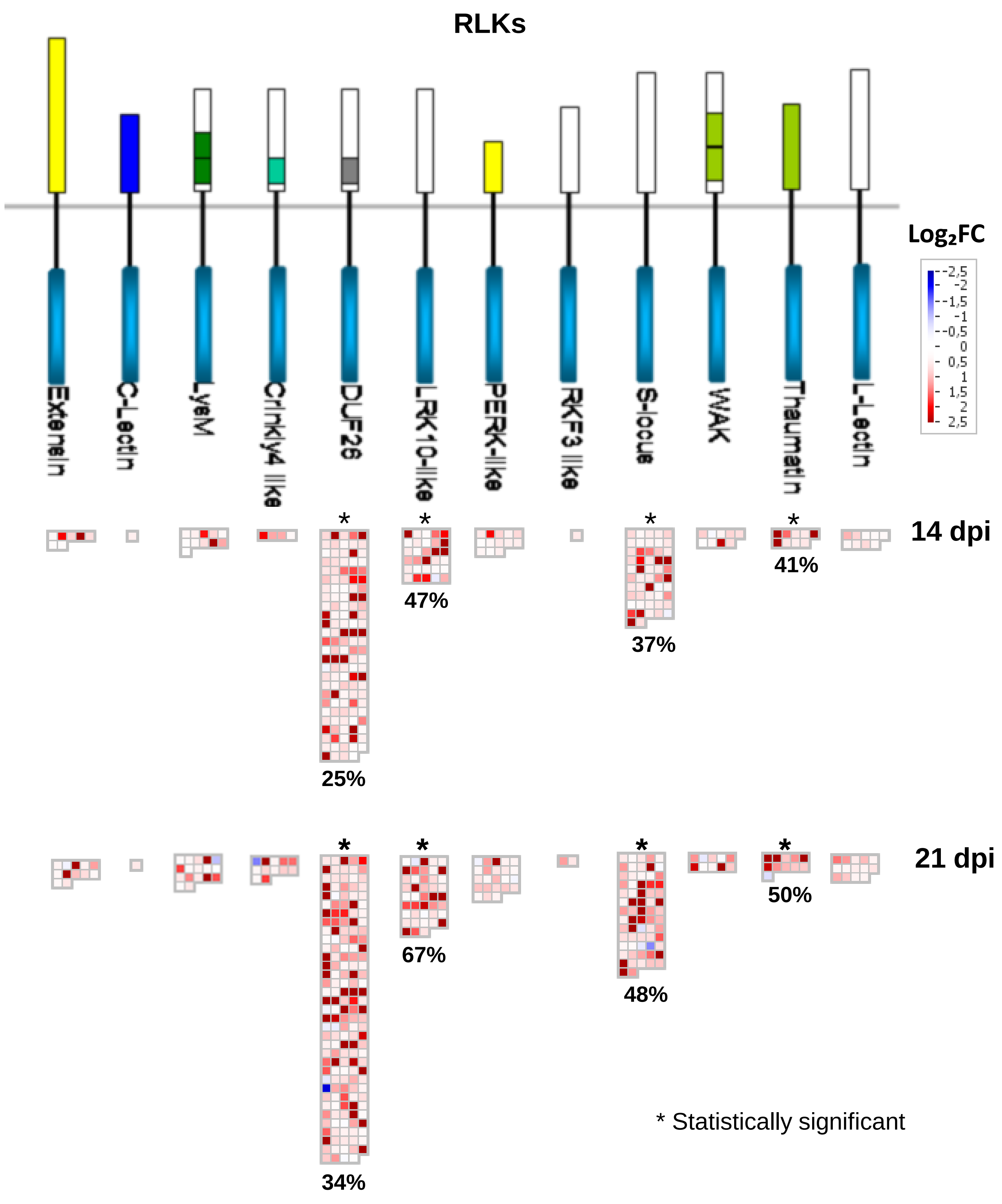

Additional file 4: FigS4A

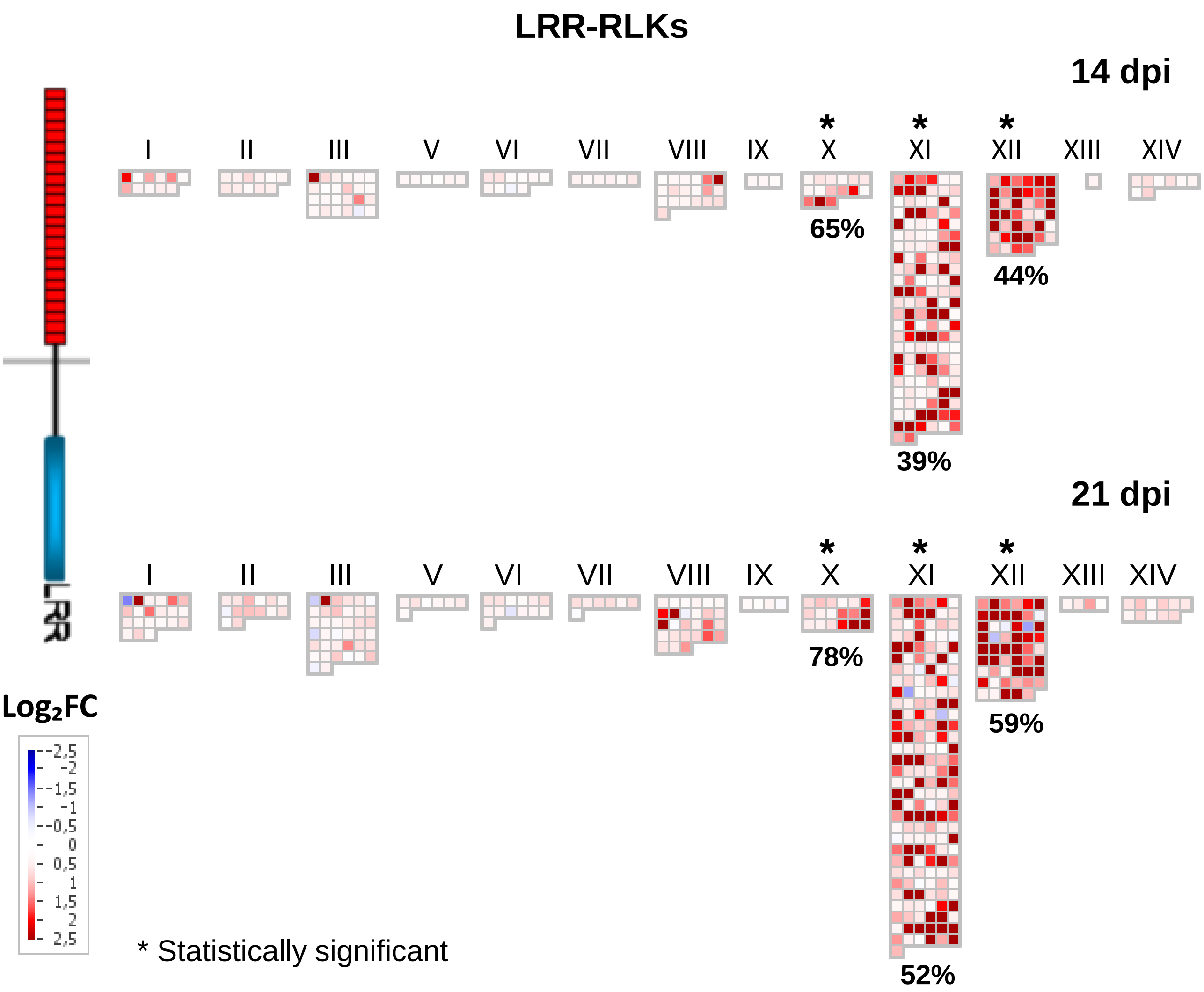

Additional file 4: FigS4B

RLCKs

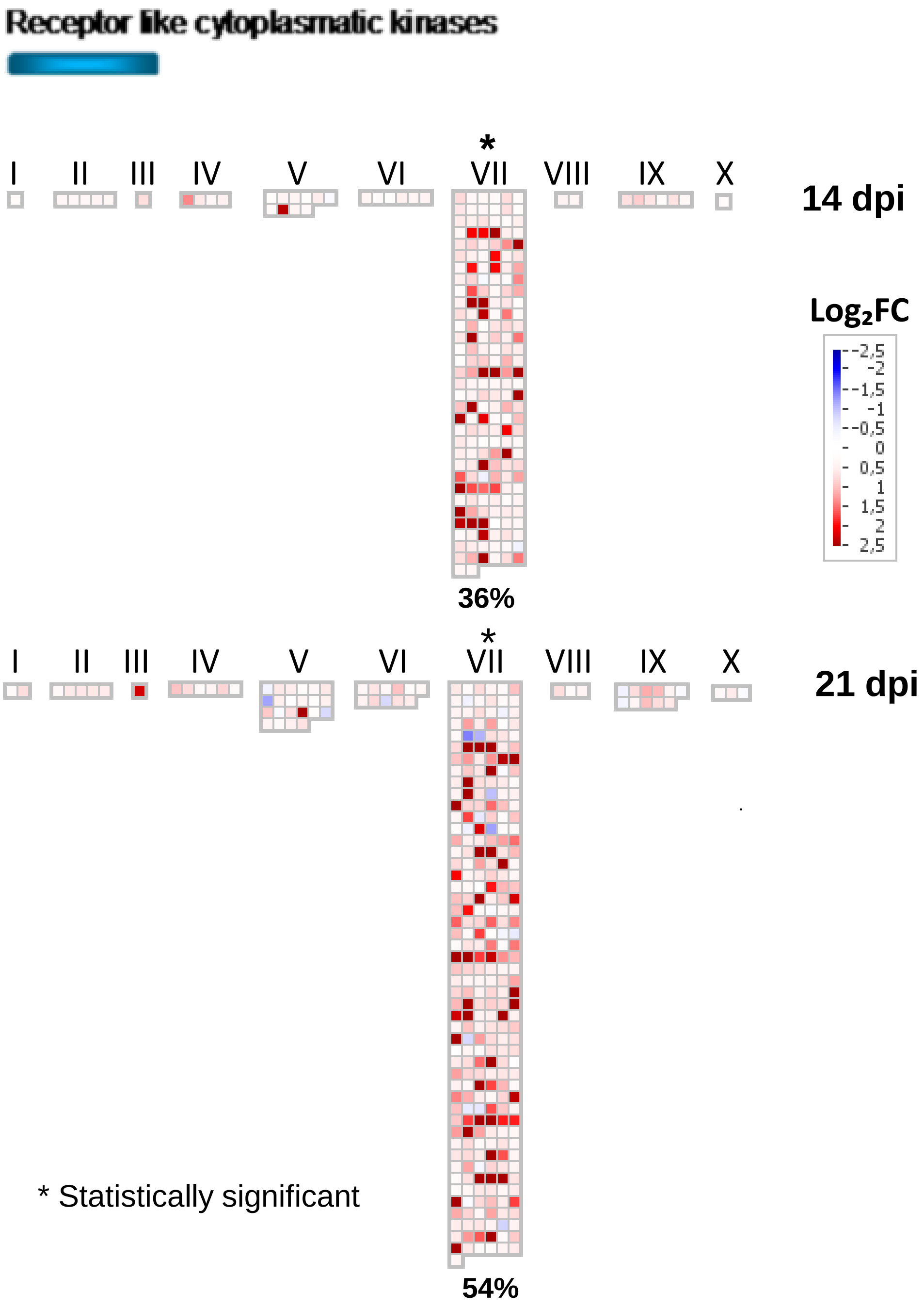

* Statistically significant

Additional file 4: FigS4C

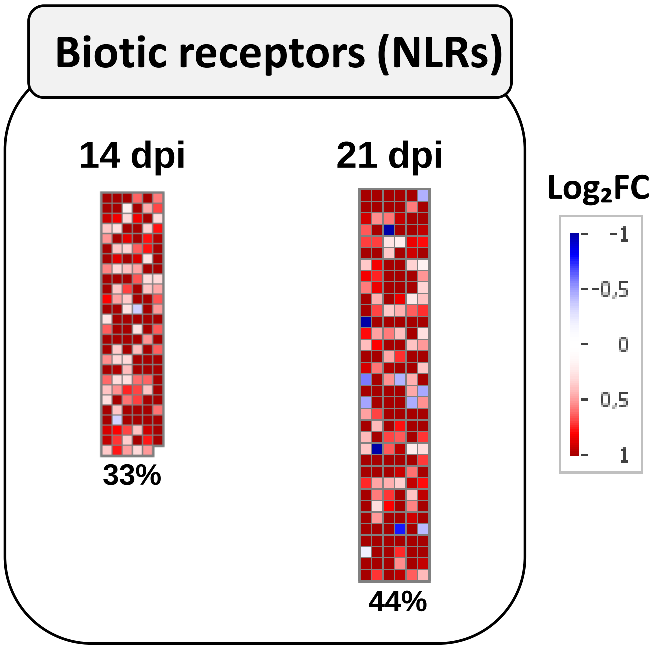

Just statistically significant categories are shown

Additional file 4: FigS4D

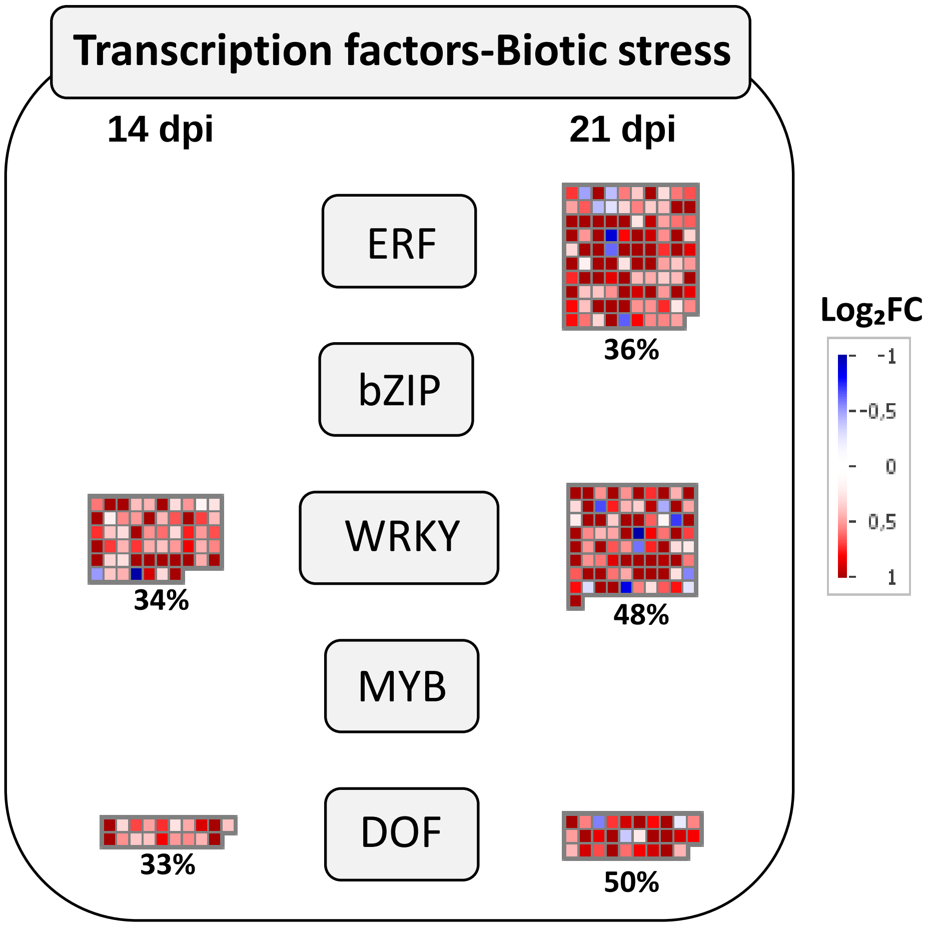

Just statistically significant categories are shown

Additional file 4: FigS4E

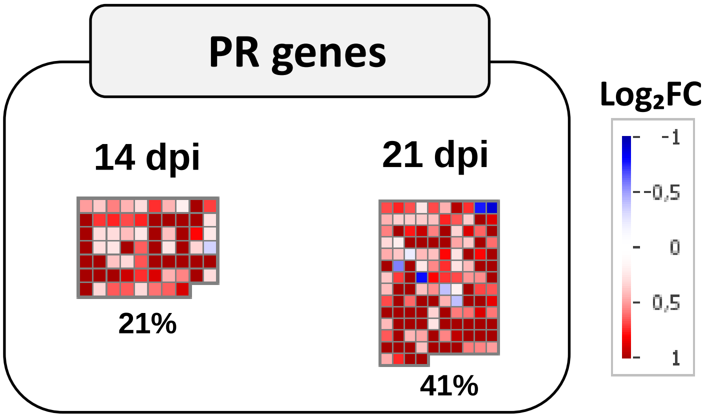

Just statistically significant categories are shown

Additional file 4: FigS4F

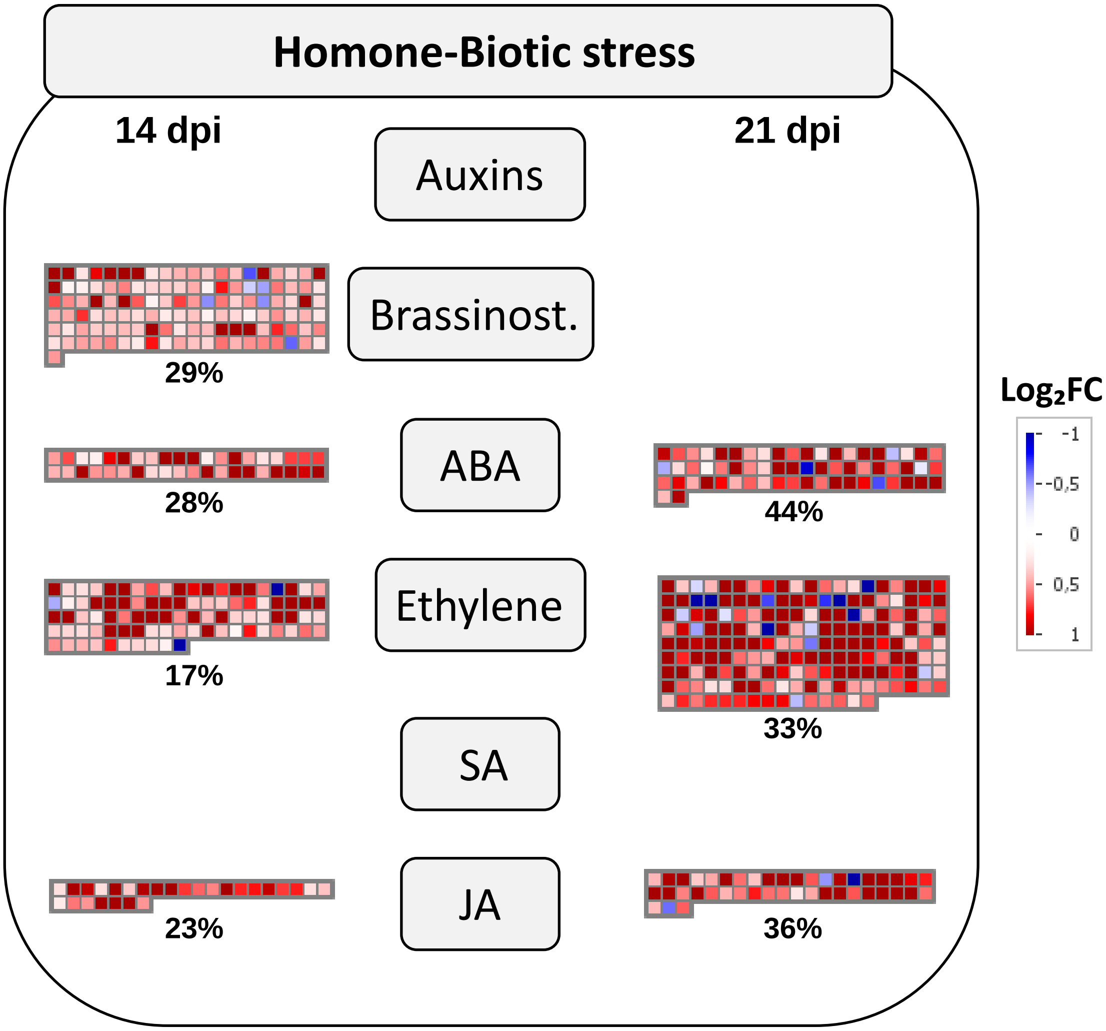

Additional file 4: FigS4G

Additional file 4: Fig. S4. Immunity-related categories (MapMan ontology) enriched in upregulated genes in response to TYLCV infection at 14 and 21 dpi. A) Receptor-like kinases (RLKs) (BIN 30.2 signaling.receptor kinases). B) Leucine rich RLKs (LRR-RLKs) (BIN 30.2 signaling.receptor kinases). Classes IV and XV from LRR-RLKs are excluded in MapMan representation. C) Receptor like cytoplasmic kinases (RLCK) (BIN 29.4.1 protein.postranslational modification.kinase). D) Biotic receptors NLR proteins (BIN 20.1.2 stress.biotic.receptors). E) Biotic stress-related transcription factors (BIN 27.3 RNA.regulation of transcription). F) Pathogenesis-related (PR) proteins proteins (BIN 20.1.7 stress.biotic.PR-proteins). G) Biotic stress-related homones (BIN 17 hormone metabolism). Only statistically significant MapMan BINs are shown in D) E) F) and G). Statistically significant MapMan BINs are identified with a * in A), B) and C) (Wilcoxon test and Benjamini-Hochberg correction adjusted p-values ≤ 0.05). Each square corresponds to the Log_2FC of a DEG, and the color represents its upregulation (red) or downregulation (blue). The percentage of gene enrichment for each MapMan ontology term is shown.
