# Supplementary material for: Transcriptional and epigenetic changes during tomato yellow leaf curl virus infection in tomato
Source: BMC Plant Biol. 2023 Dec 18;23:651. doi: 10.1186/s12870-023-04534-y (PMC10726652; doi:10.1186/s12870-023-04534-y)
Supplement: Supplementary file 5 — Additional file 5. Fig. S5. Expression profiles of the tomato gene clusters during TYLCV infection. [file 12870_2023_4534_MOESM5_ESM.pdf]

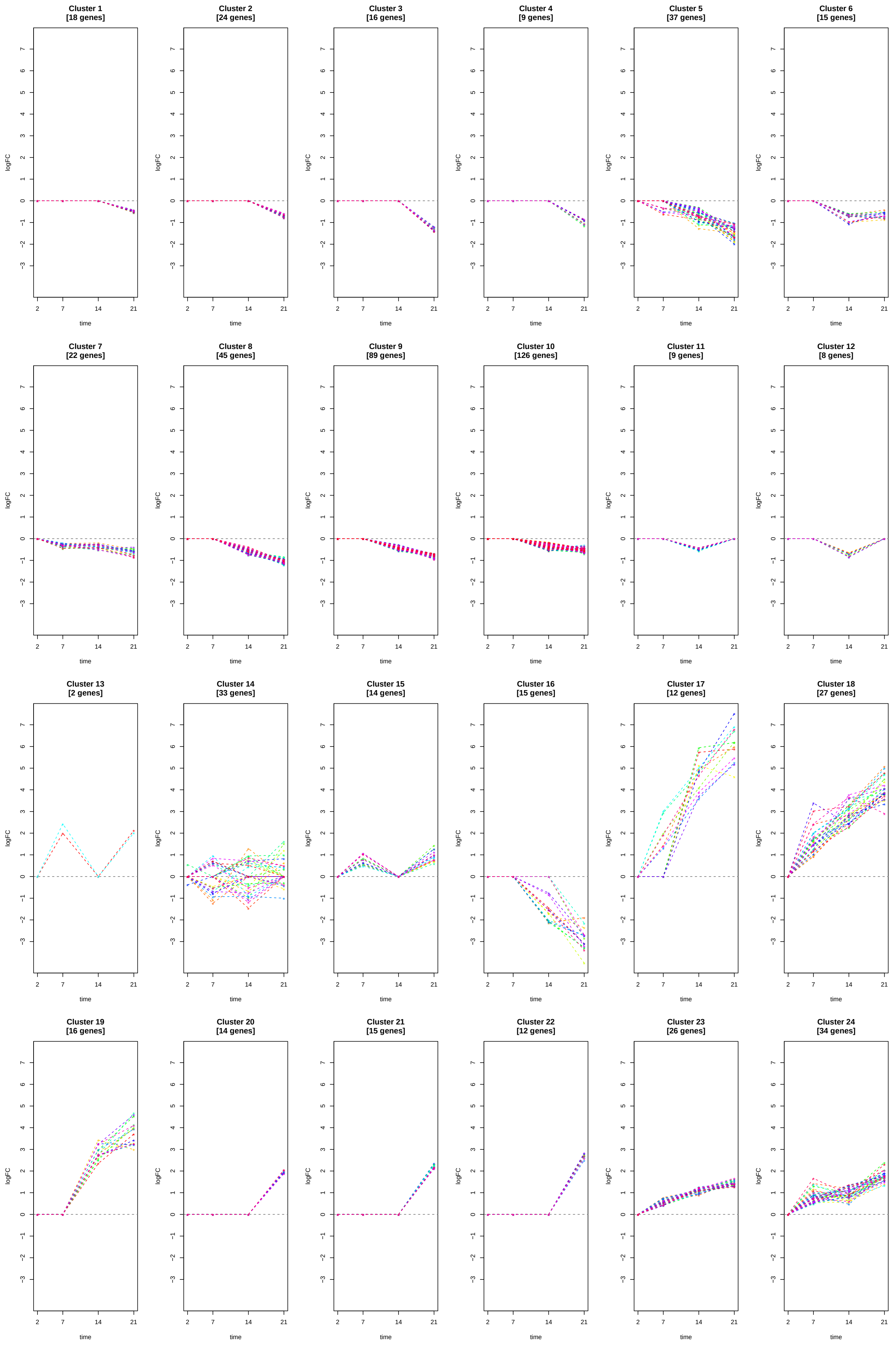
Cluster 1
[18 genes]
Cluster 2
[24 genes]
Cluster 3
[16 genes]
Cluster 4
[9 genes]
Cluster 5
[37 genes]
Cluster 6
[15 genes]
Cluster 7
[22 genes]
Cluster 8
[45 genes]
Cluster 9
[89 genes]
Cluster 10
[126 genes]
Cluster 11
[9 genes]
Cluster 12
[8 genes]
Cluster 13
[2 genes]
Cluster 14
[33 genes]
Cluster 15
[14 genes]
Cluster 16
[15 genes]
Cluster 17
[12 genes]
Cluster 18
[27 genes]
Cluster 19
[16 genes]
Cluster 20
[14 genes]
Cluster 21
[15 genes]
Cluster 22
[12 genes]
Cluster 23
[26 genes]
Cluster 24
[34 genes]
logFC
time
2
7
14
21

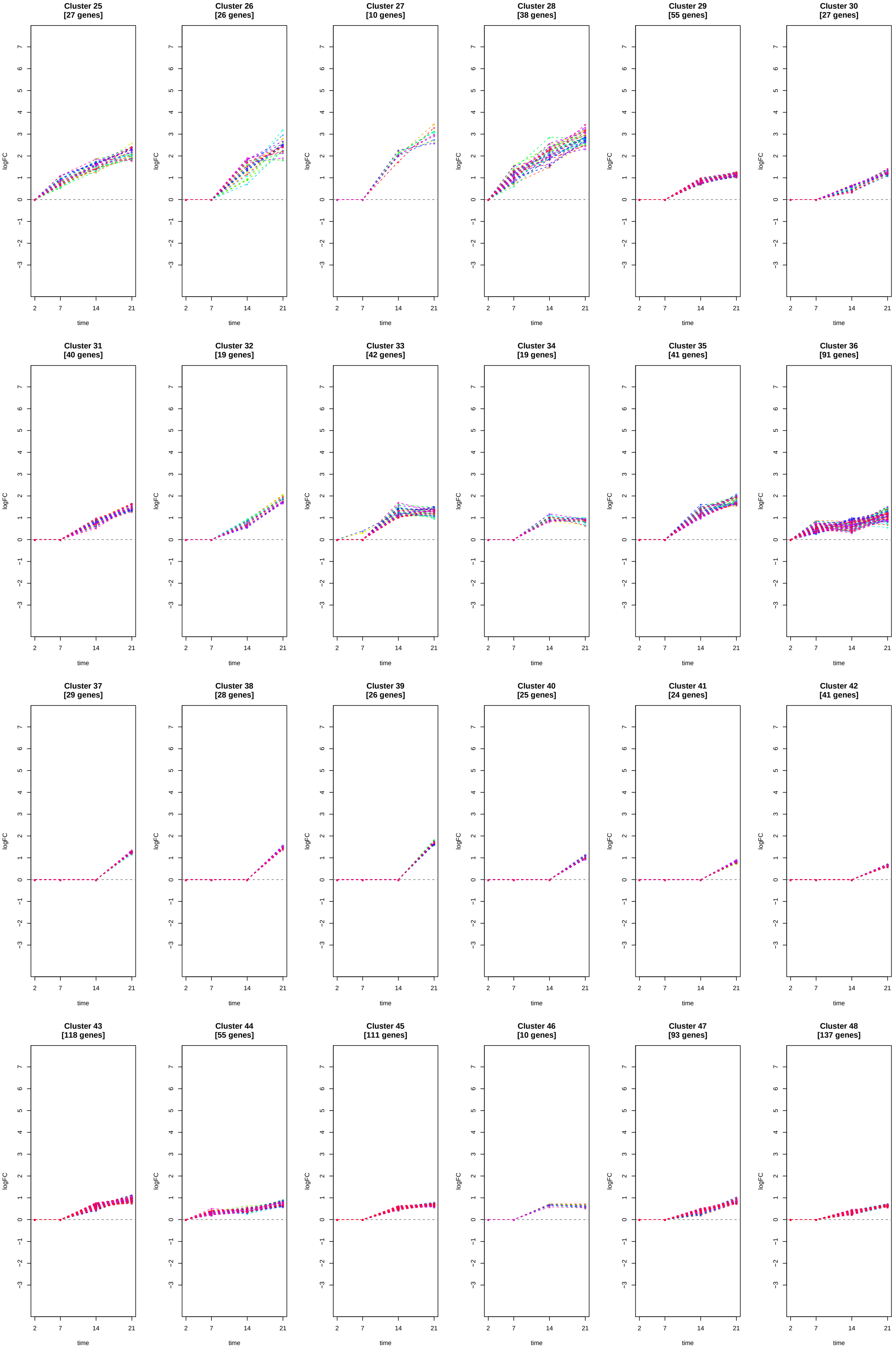

Additional file 5: Fig. S5. Expression profiles of the tomato gene clusters during TYLCV infection. DEGs were clustered into 48 groups based on their expression changes during infection. For each cluster the $\log_2$FC of the DEGs (y axis) are represented during TYLCV infection (x axis, 2, 7, 14 and 21 dpi)**.**
