# Supplementary material for: Transcriptional and epigenetic changes during tomato yellow leaf curl virus infection in tomato
Source: BMC Plant Biol. 2023 Dec 18;23:651. doi: 10.1186/s12870-023-04534-y (PMC10726652; doi:10.1186/s12870-023-04534-y)
Supplement: Supplementary file 6 — Additional file 6. Fig. S6. Functional enrichment of the tomato gene clusters. [file 12870_2023_4534_MOESM6_ESM.pdf]

A

Upregulated genes at 7, 14 and 21 dpi PANTHER GO Slim Biological Process	*GO terms*	*Ref-list (34652)*	*UP genes*	*Expect.*	*Fold Enrich.*	*raw P-value*	*FDR*
Defense response	GO:0006952	188	8	1.14	7.02	2.69E-05	5.72E-03
Gene silencing by RNA	GO:0031047	42	4	0.25	15.72	1.71E-04	2.54E-02

B

Upregulated genes at 7, 14 and 21 dpi
Mapman ontology

BIN	*BIN name*	*UP genes*	*p-value*
20.1	stress.biotic	43	0.047
20.1.2	stress.biotic.receptors	25	0.013
30	signalling	49	0.047
30.2	signalling.receptor kinases	34	0.022
30.2.11	signalling.receptor kinases.leucine rich repeat XI	19	7.91E-05
30.2.12	signalling.receptor kinases.leucine rich repeat XII	8	0.047

C

Upregulated genes at 14 and 21 dpi PANTHER GO Slim Biological Process	*GO terms*	*Ref-list (34652)*	*UP genes*	*Expect.*	*Fold Enrich.*	*raw P-value*	*FDR*
Protein autophosphorylation	GO:0046777	75	8	1.49	5.37	2.17E-04	1.40E-02
Protein glycosylation	GO:0006486	42	7	0.83	8.39	4.34E-05	5.38E-03
Protein N-linked glycosylation	GO:0006487	33	5	0.66	7.63	8.13E-04	3.18E-02
Signal transduction	GO:0007165	404	26	8.02	3.24	4.51E-07	1.12E-04
Localization	GO:0051179	1160	44	23.03	1.91	7.20E-05	6.29E-03
Establishment of localization	GO:0051234	1019	36	20.23	1.78	1.36E-03	4.49E-02
Cellular localization	GO:0051641	579	25	11.50	2.17	4.76E-04	2.08E-02

D

Downregulated genes at 14 and 21 dpi PANTHER GO Slim Biological Process	*GO terms*	*Ref-list (34652)*	*DW genes*	*Expect.*	*Fold Enrich.*	*raw P-value*	*FDR*
Alpha-amino acid metabolic process	GO:1901605	156	7	1.27	5.51	3.78E-04	1.44E-02
Cellular homeostasis	GO:0019725	92	5	0.75	6.68	1.16E-03	3.76E-02
Cellular response to oxidative stress	GO:0034599	34	4	0.28	14.46	2.47E-04	1.22E-02
Glucose metabolic process	GO:0006006	35	7	0.28	24.58	4.39E-08	8.16E-06
Glycolytic process	GO:0006096	32	4	0.26	15.36	2.00E-04	1.06E-02
Hexose biosynthetic process	GO:0019319	18	4	0.15	27.31	2.71E-05	2.24E-03
Photosynthesis	GO:0015979	44	13	0.36	36.31	8.35E-16	1.24E-12
Plastid organization	GO:0009657	56	4	0.46	8.78	1.42E-03	4.40E-02
Response to light stimulus	GO:0009416	89	11	0.72	15.19	5.85E-10	2.17E-07

Additional file 6: Fig. S6. Functional enrichment of the tomato gene clusters. A) Gene ontology (GO) terms enriched in upregulated genes at 7, 14 and 21 dpi (corresponding to gene clusters 17,18,23,24,25,28,36) (255 genes). B) MapMan ontology of upregulated genes at 7, 14 and 21 dpi (corresponding to gene clusters 17,18,23,24,25,28,36) (255 genes). C) GO enrichment of upregulated genes at 14 and 21 dpi (corresponding to gene clusters 19 ,26 ,27 ,29 ,30 ,31 ,32 ,33 ,34 ,35, 43 ,45 ,46 ,47 ,48) (764 genes). D) GO enrichment of downregulated genes at 14 and 21 dpi (corresponding to gene clusters 5,6,8,9,10,16) (327 genes). GO analysis using the PANTHER GO-Slim biological process (statistical overrepresentation test). Selected statistically significant biological processes are shown (FDR adjusted p-value ≤0.05) for A), C) and D). MapMan ontology analysis in B) shows selected statistically significant MapMan BINs (Wilcoxon test and Benjamini-Hochberg FDR adjusted p-values ≤0.05).
