# Supplementary material for: Transcriptional and epigenetic changes during tomato yellow leaf curl virus infection in tomato
Source: BMC Plant Biol. 2023 Dec 18;23:651. doi: 10.1186/s12870-023-04534-y (PMC10726652; doi:10.1186/s12870-023-04534-y)
Supplement: Supplementary file 7 — Additional file 7. Fig. S7. Differentially expressed tomato siRNA loci (DEsiRNAs) overlapped with gene promoters, gene bodies and TEs/repeats during TYLCV infection. [file 12870_2023_4534_MOESM7_ESM.pdf]

A

DEsiRNAs	***7 dpi*** 21-nt	22-nt	24-nt	***14 dpi*** 21-nt	22-nt	24-nt	***21 dpi*** 21-nt	22-nt	24-nt
promoter	0	1	60	11	2	29	30	10	629
gene body	2	1	35	24	2	15	65	14	438
TE/repeats	20	117	233	22	111	87	53	193	1162

B

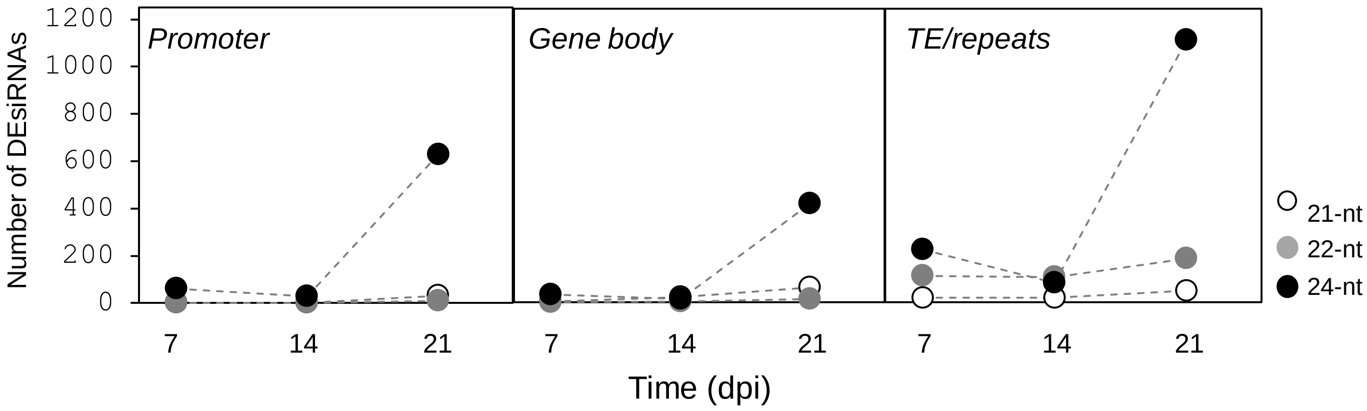

Additional file 7: Fig. S7. Differentially expressed tomato siRNA loci (DEsiRNAs) overlapped with gene promoters, gene bodies and TE/repeats during TYLCV infection. (A) Number of the unique 21-, 22- and 24-nt DEsiRNAs (FDR adjusted p-value < 0.05) that were mapped at promoters (2 kb upstream of transcriptional start site), gene bodies and TE/repeats at 7, 14 and 21 dpi. (B) Dynamic of the 21-, 22- and 24-nt DEsiRNAs that were mapped at promoters, gene bodies and TEs/repeats at 7, 14 and 21 dpi.
