# Supplementary material for: Transcriptional and epigenetic changes during tomato yellow leaf curl virus infection in tomato
Source: BMC Plant Biol. 2023 Dec 18;23:651. doi: 10.1186/s12870-023-04534-y (PMC10726652; doi:10.1186/s12870-023-04534-y)
Supplement: Supplementary file 8 — Additional file 8. Fig. S8. Tomato miRNA families. [file 12870_2023_4534_MOESM8_ESM.pdf]

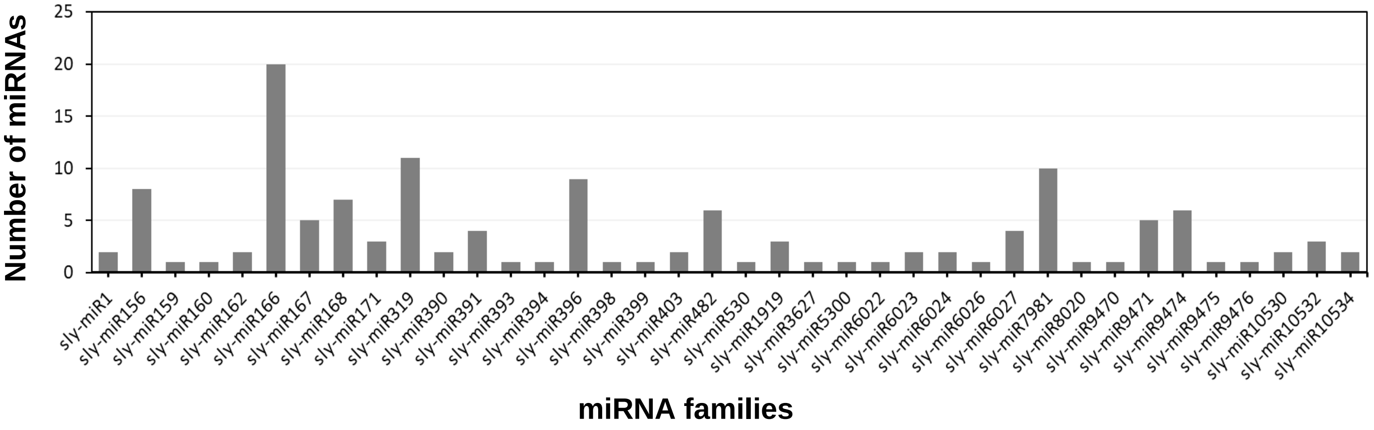

Additional file 8: Fig. S8. Tomato miRNA families. Number of unique miRNAs (y axis) from different miRNA families (x axis) are shown detected in the tomato samples (naïve, mock and TYLCV-infected at any dpi). Only miRNAs with at least 10 CPM in both biological replicates in any of the samples were considered.
