# Supplementary material for: Transcriptional and epigenetic changes during tomato yellow leaf curl virus infection in tomato
Source: BMC Plant Biol. 2023 Dec 18;23:651. doi: 10.1186/s12870-023-04534-y (PMC10726652; doi:10.1186/s12870-023-04534-y)
Supplement: Supplementary file 9 — Additional file 9. Fig. S9. Functional enrichment of the predicted target genes from the DEmiRNAs in response to TYLCV infection at 21 dpi. [file 12870_2023_4534_MOESM9_ESM.pdf]

**A**

*Predicted target genes of DEmiRNA at 21 dpi (TYLCV/mock)*

| *PANTHER GO Slim Biological Process* | *GO terms* | *Ref-list (34652)* | *Genes* | *Expect.* | *Fold Enrich.* | *raw P-value* | *FDR* |
|---|---|---|---|---|---|---|---|
| Response to stress | GO:0006950 | 662 | 43 | 6.78 | 6.34 | 5.09E-21 | 9.45E-19 |
| Defense response | GO:0006952 | 188 | 38 | 1.93 | 19.73 | 8.45E-35 | 1.79E-32 |
| Defense response to other organism | GO:0098542 | 146 | 38 | 1.50 | 25.41 | 2.21E-38 | 8.19E-36 |
| Multicellular organism development | GO:0007275 | 121 | 9 | 1.24 | 7.26 | 7.69E-06 | 5.71E-04 |
| System development | GO:0048731 | 95 | 9 | 0.97 | 9.25 | 1.21E-06 | 9.97E-05 |
| Shoot system development | GO:0048367 | 69 | 9 | 0.71 | 12.73 | 1.01E-07 | 8.83E-06 |
| Developmental process | GO:0032502 | 196 | 9 | 2.01 | 4.48 | 2.66E-04 | 1.72E-02 |
| Anatomical structure morphogenesis | GO:0009653 | 65 | 9 | 0.67 | 13.52 | 6.34E-08 | 5.89E-06 |
| Multicellular organismal process | GO:0032501 | 124 | 9 | 1.27 | 7.08 | 9.25E-06 | 6.55E-04 |
| Morphogenesis of a branching structure | GO:0001763 | 18 | 8 | 0.18 | 43.38 | 1.38E-10 | 1.71E-08 |

**B**

*Predicted target genes of DEmiRNA at 21 dpi (TYLCV/mock)*
*Mapman ontology*

| *BIN* | *BIN name* | *DEGs* | *p-value* |
|---|---|---|---|
| ***20*** | stress | 43 | 0.006 |
| ***20.1*** | stress.biotic | 41 | 0.001 |
| ***30*** | signalling | 18 | 0.029 |
| ***30.2*** | signalling.receptor kinases | 15 | 0.009 |
| ***30.2.11*** | signalling.receptor kinases.leucine rich repeat XI | 14 | 0.009 |

**Additional file 9: Fig. S9. Functional enrichment of the predicted target genes from the DEmiRNAs in response to TYLCV infection at 21 dpi.** A) Gene ontology (GO) analysis using the PANTHER GO-Slim biological process (statistical overrepresentation test) of all the predicted targets from DEmiRNAs at 21 dpi (684 miRNAs-targets pairs, 470 unique targets). Selected statistically significant biological processes are shown (FDR adjusted p-value ≤ 0.05). B) MapMan ontology of predicted DE targets of DEmiRNAs at 21 dpi (352 miRNAs-targets pairs, 235 unique targets). Selected statistically significant MapMan BINs are shown (Wilcoxon test and Benjamini-Hochberg FDR adjusted p-values ≤0.05).
