# Supplementary material for: Transcriptional and epigenetic changes during tomato yellow leaf curl virus infection in tomato
Source: BMC Plant Biol. 2023 Dec 18;23:651. doi: 10.1186/s12870-023-04534-y (PMC10726652; doi:10.1186/s12870-023-04534-y)
Supplement: Supplementary file 10 — Additional file 10. Fig. S10. Expression levels of the DEmiRNAs and their predicted target genes in tomato according to the degradome analysis. [file 12870_2023_4534_MOESM10_ESM.pdf]

A

| Number of miRNA-target pairs (PARE) | | | |
|---|---|---|---|
| ***miRNA*** | ***Target*** | ***14 dpi*** | ***21 dpi*** |
| **DW** | **UP** | 1 | 17 (39%) |
| **UP** | **DW** | 0 | 0 |
| **DW** | **DW** | 0 | 0 |
| **UP** | **UP** | 0 | 12 (28%) |
| **DW** | **nc** | 1 | 9 (21%) |
| **UP** | **nc** | 1 | 5 (12%) |
| **TOTAL** | | 3 | 43 (100%) |

B

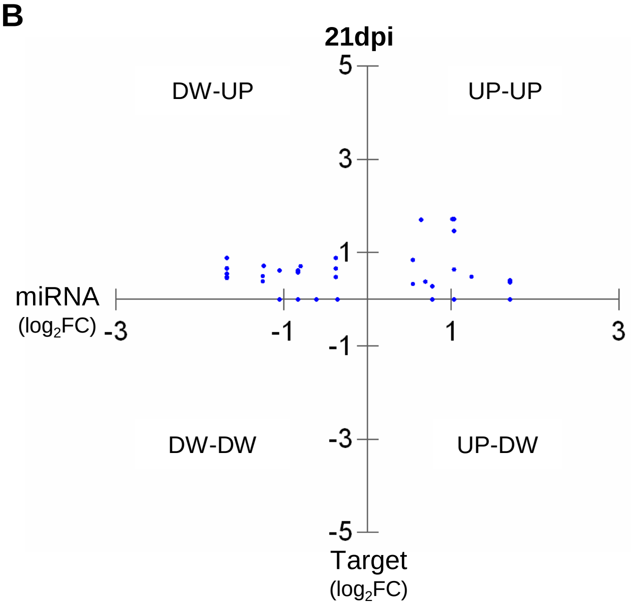


**Additional file 10: Fig. S10. Expression levels of the DEmiRNAs and their predicted target genes in tomato according to the degradome analysis.** A) Classification based on their expression levels at 14 and 21 dpi of the miRNA-target pairs according to degradome´s data [82]. UP: upregulation, DW: downregulation, nc (no changes): target gene not differentially expressed. B) Expression level ($log_2FC$ for the ratio TYLCV/mock) at 21 dpi of the DEmiRNAs (x axis) and their target genes (y axis). Only targets with a degradome category ≤ 3 and a p-value ≤ 0.05 are shown.
