# Supplementary material for: Transcriptional and epigenetic changes during tomato yellow leaf curl virus infection in tomato
Source: BMC Plant Biol. 2023 Dec 18;23:651. doi: 10.1186/s12870-023-04534-y (PMC10726652; doi:10.1186/s12870-023-04534-y)
Supplement: Supplementary file 11 — Additional file 11. Fig. S11. Functional enrichment of putative targets of the DEphasiRNAs upon TYLCV infection at 21 dpi. [file 12870_2023_4534_MOESM11_ESM.pdf]

A

Predicted target genes of DEphasiRNA at 21 dpi (TYLCV/mock) PANTHER GO. Slim Biological Process	GO terms	Ref-list (34652)	Genes	Expect.	Fold enrich.	raw P-value	FDR
Response to stress	GO:0006950	662	99	28.45	3.48	3.97E-24	1.97E-21
Defence response	GO:0006952	188	48	8.08	5.94	3.24E-20	4.38E-18
Defence response to other organism	GO:0098542	146	46	6.27	7.33	1.59E-22	2.94E-20
Negative regulation of gene expression	GO:0010629	131	15	5.63	2.66	1.06E-03	2.71E-02
Post-transcriptional gene silencing by RNA	GO:0035194	42	8	1.80	4.43	9.36E-04	2.53E-02
Cell cycle	GO:0007049	264	26	11.34	2.29	2.54E-04	1.18E-02
Mitotic cell cycle	GO:0000278	191	19	8.21	2.32	1.32E-03	3.21E-02
Mitotic sister chromatid segregation	GO:0000070	47	8	2.02	3.96	1.77E-03	3.99E-02
Mitotic sister chromatid cohesion	GO:0007064	21	6	0.90	6.65	6.80E-04	2.30E-02
Cell wall biogenesis	GO:0042546	72	10	3.09	3.23	1.97E-03	4.19E-02
Cell division	GO:0051301	37	7	1.59	4.40	2.00E-03	4.19E-02
ph reduction	GO:0045851	6	5	0.26	19.39	4.42E-05	3.29E-03
Vacuolar acidification	GO:0007035	6	5	0.26	19.39	4.42E-05	3.13E-03
Localization	GO:0051179	1160	75	49.85	1.50	9.88E-04	2.58E-02
Establishment of localization	GO:0051234	1019	70	43.79	1.60	2.57E-04	1.16E-02
Transport	GO:0006810	1005	70	43.18	1.62	1.72E-04	9.13E-03

B

Predicted target genes of DEphasiRNA at 21 dpi (TYLCV/mock)
Mapman ontology

BIN	BIN name	DEGs	p-value
20	stress	122	3.55E-07
20.1	stress.biotic	77	6.14E-11
20.1.2	stress.biotic.receptors	57	3.55E-11
26	misc	135	0.002
26.12	misc.peroxidases	12	0.004
29.4.1.57	protein.postranslational modification.kinase.receptor like cytoplasmatic kinase VII	28	0.013
30	signalling	136	1.09E-05
30.2	signalling.receptor kinases	71	6.14E-11
30.2.11	signalling.receptor kinases.leucine rich repeat XI	38	1.21E-08
30.2.12	signalling.receptor kinases.leucine rich repeat XII	18	3.86E-05
30.2.17	signalling.receptor kinases.DUF 26	19	0.001
30.2.24	signalling.receptor kinases.S-locus glycoprotein like	11	0.002

Additional file 11: Fig. S11. Functional enrichment of putative targets of the DEphasiRNAs upon TYLCV infection at 21 dpi. A) Gene ontology (GO) analysis using the PANTHER GO-Slim biological process (statistical overrepresentation test) of all the predicted targets for the DEphasiRNA at 21 dpi (2697 DEphasiRNAs-target pairs, 2154 unique targets). Selected statistically significant biological process are shown (FDR adjusted p-value ≤ 0.05). B) MapMan ontology of predicted DE targets of DEphasiRNA at 21 dpi (1097 phasiRNAs-targets pairs, 923 unique targets). Selected statistically significant MapMan BINs are shown (Wilcoxon test and Benjamini-Hochberg correction adjusted p-values ≤ 0.05).
