# Supplementary material for: Transcriptional and epigenetic changes during tomato yellow leaf curl virus infection in tomato
Source: BMC Plant Biol. 2023 Dec 18;23:651. doi: 10.1186/s12870-023-04534-y (PMC10726652; doi:10.1186/s12870-023-04534-y)
Supplement: Supplementary file 12 — Additional file 12. Fig. S12. Expression levels of the DE phasiRNAs and their predicted target genes in tomato according to the degradome analysis. [file 12870_2023_4534_MOESM12_ESM.pdf]

A

Number of phasiRNA-target pairs (PARE)			
phasiRNA	***Target***	***14dpi***	***21 dpi***
DW	***UP***	0	40 (31%)
UP	***DW***	3	12 (9%)
DW	***DW***	0	10 (8%)
UP	***UP***	6	37 (29%)
DW	***nc***	1	18 (14%)
UP	***nc***	7	12 (9%)
TOTAL		17	129 (100%)

B

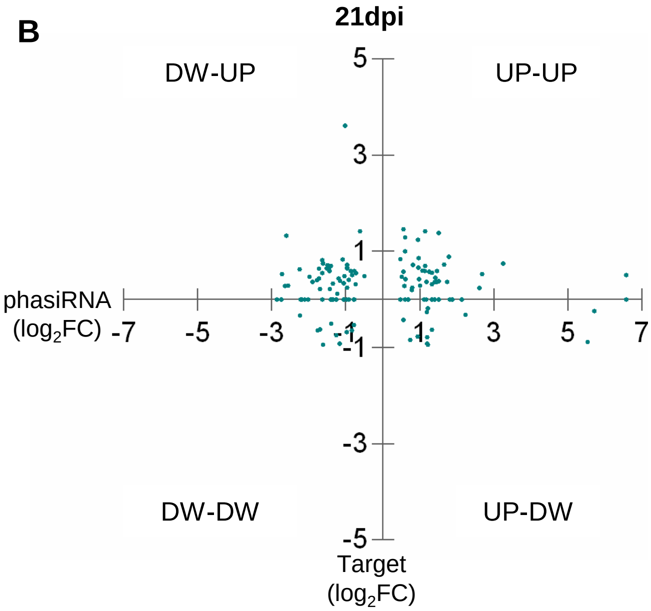

Additional file 12: Fig. S12. Expression levels of the DE phasiRNAs and their predicted target genes in tomato according to the degradome analysis. A) Classification based on their expression levels at 14 and 21 dpi of the phasiRNA-target pairs according to degradome´s data [82]. UP: upregulation, DW: downregulation, nc (no changes): target gene not differentially expressed. B) Expression level (log_2FC for the ratio TYLCV/mock) at 21 dpi of the DEphasiRNAs (x axis) and their target genes (y axis). Only targets with a degradome category ≤ 3 and a p-value ≤ 0.05 are shown.
