# Supplementary material for: Transcriptional and epigenetic changes during tomato yellow leaf curl virus infection in tomato
Source: BMC Plant Biol. 2023 Dec 18;23:651. doi: 10.1186/s12870-023-04534-y (PMC10726652; doi:10.1186/s12870-023-04534-y)
Supplement: Supplementary file 13 — Additional file 13. Fig. S13. Tomato miRNA-PHAS loci-phasiRNA network upon TYLCV infection at 14 dpi. [file 12870_2023_4534_MOESM13_ESM.pdf]

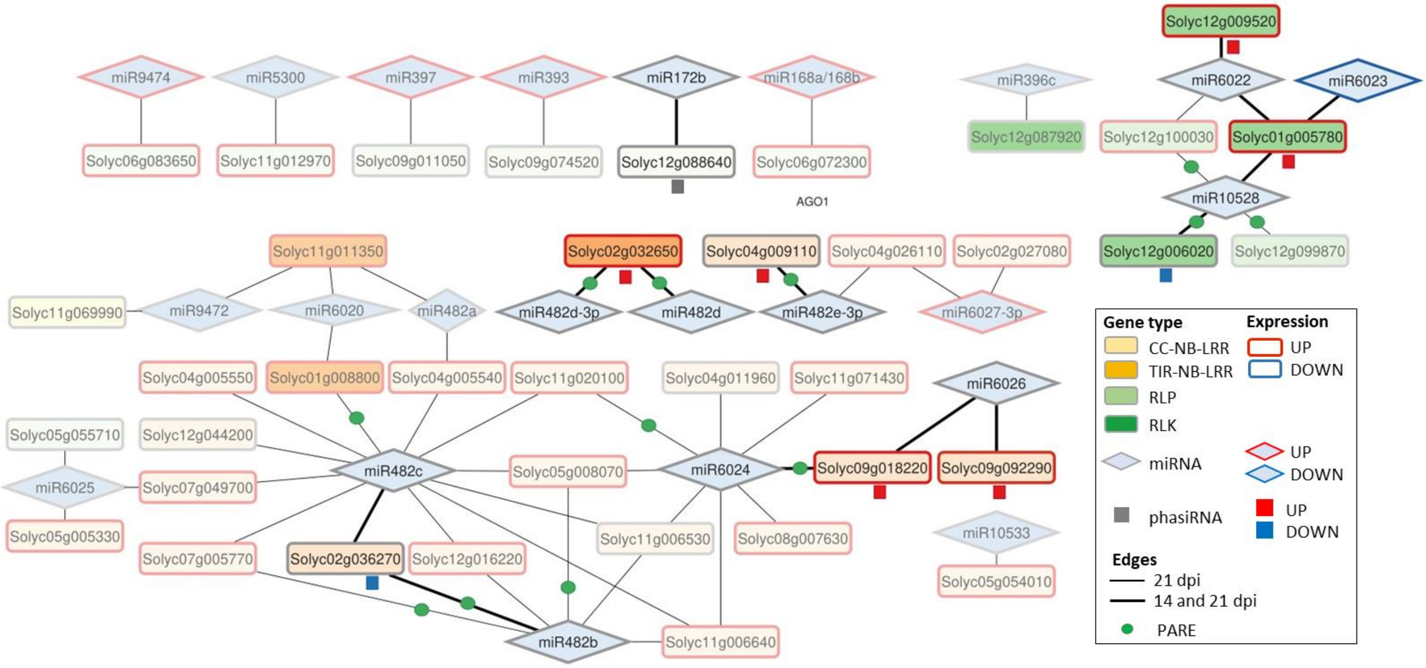

Additional file 13: Fig. S13. Tomato miRNA-*PHAS* loci-phasiRNA network upon TYLCV infection at 14 dpi. Network representation of the miRNAs (21 and 22-nt, rhombus) that trigger the formation of DEphasiRNAs (squares) from their target *PHAS* loci transcripts (rectangles) at 14 dpi. Each geometrical form is surrounded by a colored line that indicates their differential expression pattern: red for induced, blue for repressed and grey when they are not differentially expressed. The miRNA isoform that triggers each *PHAS* loci is indicated in Dataset S7. The different types of *PHAS* loci are marked by colors and indicated in the figure legend. The black edges connect a miRNA trigger and a targeting *PHAS* locus, the thick ones indicate that the pair was also observed at 21 dpi (Fig. 8) and the interactions that just take place at 21 dpi are shaded. miRNA-*PHAS* locus pairs that have also been identified using the degradome analysis are marked with a green circle.
