# Supplementary material for: Transcriptional and epigenetic changes during tomato yellow leaf curl virus infection in tomato
Source: BMC Plant Biol. 2023 Dec 18;23:651. doi: 10.1186/s12870-023-04534-y (PMC10726652; doi:10.1186/s12870-023-04534-y)
Supplement: Supplementary file 14 — Additional file 14. Fig. S14. Percentage of DNA methylation at the three cytosine contexts (CG, CHG, CHH). [file 12870_2023_4534_MOESM14_ESM.pdf]

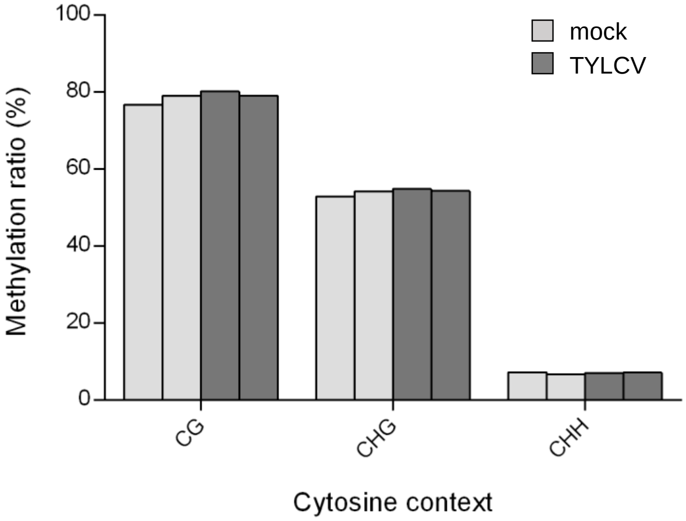

Additional file 14: Fig. S14. Percentage of DNA methylation at the three cytosine contexts (CG, CHG, CHH). Data from the two different biological replicates from TYLCV-tomato infected plants (dark grey) and control plants (mock, light grey) at 14 dpi are shown.
