# Supplementary material for: Transcriptional and epigenetic changes during tomato yellow leaf curl virus infection in tomato
Source: BMC Plant Biol. 2023 Dec 18;23:651. doi: 10.1186/s12870-023-04534-y (PMC10726652; doi:10.1186/s12870-023-04534-y)
Supplement: Supplementary file 15 — Additional file 15. Fig. S15. Dynamic of the deregulated tomato mRNAs, miRNAs and phasiRNAs during TYLCV infection. [file 12870_2023_4534_MOESM15_ESM.pdf]

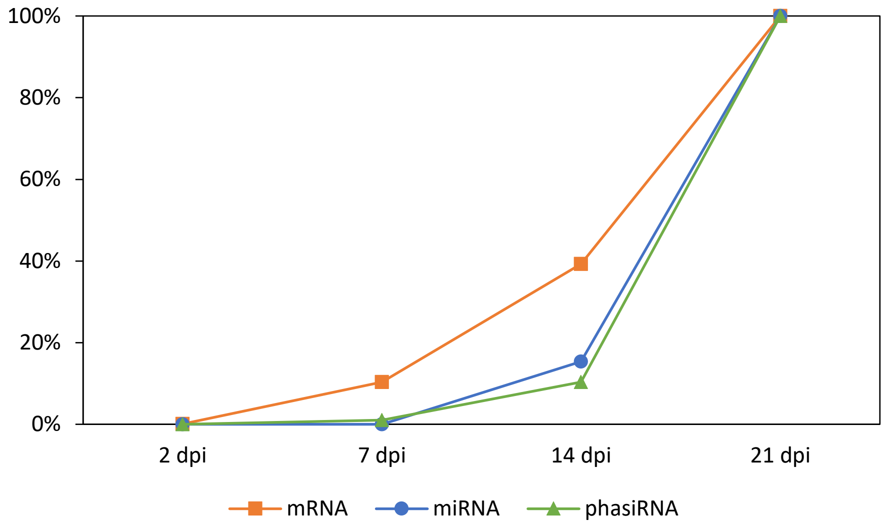

Additional file 15: Fig. S15. Dynamic of the deregulated tomato mRNAs, miRNAs and phasiRNAs during TYLCV infection. Percentage of the deregulated mRNAs (orange), miRNAs (blue) and phasiRNAs (green) at 2, 7, 14 and 21 dpi, if the total number of deregulated mRNAs (6122), miRNAs (52) and phasiRNAs (300) at 21 dpi is set to 100%.
